# Attractor Metabolic Networks

**DOI:** 10.1371/journal.pone.0058284

**Published:** 2013-03-15

**Authors:** Ildefonso M. De la Fuente, Jesus M. Cortes, David A. Pelta, Juan Veguillas

**Affiliations:** 1 Quantitative Biomedicine Unit, BioCruces Health Research Institute, Barakaldo, Basque Country, Spain; 2 Institute of Parasitology and Biomedicine “López Neyra”, CSIC, Granada, Spain; 3 Department of Mathematics, University of the Basque Country, EHU-UPV, Leioa, Basque Country, Spain; 4 Ikerbasque: The Basque Foundation for Science, Bilbao, Basque Country, Spain; 5 Department of Computer Science and Artificial Intelligence, University of Granada, Granada, Spain; Universidad de La Laguna, Spain

## Abstract

**Background:**

The experimental observations and numerical studies with dissipative metabolic networks have shown that cellular enzymatic activity self-organizes spontaneously leading to the emergence of a Systemic Metabolic Structure in the cell, characterized by a set of different enzymatic reactions always locked into active states (metabolic core) while the rest of the catalytic processes are only intermittently active. This global metabolic structure was verified for *Escherichia coli*, *Helicobacter pylori* and *Saccharomyces cerevisiae*, and it seems to be a common key feature to all cellular organisms. In concordance with these observations, the cell can be considered a complex metabolic network which mainly integrates a large ensemble of self-organized multienzymatic complexes interconnected by substrate fluxes and regulatory signals, where multiple autonomous oscillatory and quasi-stationary catalytic patterns simultaneously emerge. The network adjusts the internal metabolic activities to the external change by means of flux plasticity and structural plasticity.

**Methodology/Principal Findings:**

In order to research the systemic mechanisms involved in the regulation of the cellular enzymatic activity we have studied different catalytic activities of a dissipative metabolic network under different external stimuli. The emergent biochemical data have been analysed using statistical mechanic tools, studying some macroscopic properties such as the global information and the energy of the system. We have also obtained an equivalent Hopfield network using a Boltzmann machine. Our main result shows that the dissipative metabolic network can behave as an attractor metabolic network.

**Conclusions/Significance:**

We have found that the systemic enzymatic activities are governed by attractors with capacity to store functional metabolic patterns which can be correctly recovered from specific input stimuli. The network attractors regulate the catalytic patterns, modify the efficiency in the connection between the multienzymatic complexes, and stably retain these modifications. Here for the first time, we have introduced the general concept of attractor metabolic network, in which this dynamic behavior is observed.

## Introduction

Living cells are essentially highly evolved dynamic metabolic structures, in which the more complex known molecules are synthesized and destroyed by means of a sophisticated metabolic network characterized by millions of biochemical reactions, densely integrated, shaping one of the most complex dynamic systems in nature [1], [2].

The enzymes are the main molecules of this surprising biochemical reactive network. They are responsible for almost all the biomolecular transformations, which globally considered it is called cellular metabolism.

Intensive studies of protein-protein interactions have shown that the internal cellular medium is an assembly of supra-molecular protein complexes [3], e.g., the analyses of the proteome of *Saccharomyces cerevisiae* have shown that at least 83% of all proteins form complexes (containing from two to eighty-three proteins), and their overall enzymatic structure is formed by a modular network of biochemical interactions between multienzyme complexes [4]. This molecular self-organization occurs in all kinds of cells, both eukaryotes and prokaryotes [5]–[7].

The self-organization [8] of cooperating enzymes into multienzyme complexes [9], seem to be central feature of cellular metabolism, crucial for the functional activity, regulation and efficiency of biomolecular processes and fundamental for understanding the molecular architecture of cell life (see for more details Supporting Information S1).

Apart from forming complex enzymatic associations, the catalytic dynamics of multienzymatic sets present metabolic transitions between different quasi-stationary and oscillatory molecular patterns [10] (see Supporting Information S1).

From a dynamic point of view, the multienzymatic complexes represent dissipative structures in which oscillatory patterns and functional integrative processes emerge, allowing the reactive coordination between their catalytic parts [10]. These self-organized multienzymatic complexes associated with other non-catalytic biomolecular structures are called metabolic subsystems [10].

In concordance with the structural and functional self-organization of enzymes (see Supporting Information S1), the cell can be considered a complex metabolic network which mainly integrates a large ensemble of dissipative metabolic subsystems, where multiple autonomous oscillatory and some quasi-stationary activity patterns simultaneously emerge [9], [10].

In order to research the functionality of the cellular metabolism, the dissipative metabolic networks (DMNs) were created [11], [12]. Essentially, a DMN is an open system formed by a given set of metabolic subsystems (self-organized multienzymatic complexes) interconnected by biochemical substrate fluxes and three classes of biomolecular regulatory signals: activatory (positive allosteric modulation), inhibitory (negative allosteric modulation) and all-or-nothing type (which correspond to the regulatory enzymes of covalent modulation) [13]. Therefore, each metabolic subsystem is connected with others by a structure composed of biochemical message flows [14]. This dynamic process of biochemical interconnections between subsystems may be understood as metabolic synapses i.e., the functional connection processes among self-organized multienzymatic complexes through which biomolecular information flows from one metabolic subsystem to another [14].

In the DMN, the emergent output activity (the connection pattern) for each metabolic subsystem can be either steady state or oscillatory with an infinite number of distinct activity regimes [12], [15].

The DMN adjusts the internal metabolic activities to the external environmental change by means of flux plasticity i.e., changes in the physiological values of the metabolic synapses which lead to a differential catalytic activity of subsystems, and structural plasticity i.e., persistent change in the state of the metabolic subsystems which can be active state, *on-off* changing state and inactive state: all these catalytic states are also due to the metabolic synaptic changes. Flux plasticity and structural plasticity have been experimentally observed in the metabolism of several organisms as the main systemic biomolecular mechanisms for the adaptation to external perturbations [16], [17].

Numerous mathematical studies on metabolic rhythms have contributed to a better understanding of the functionality of the self-organized multienzymatic subsystems (the nodes of the dissipative metabolic networks). Most of these functional biochemical studies have been carried out by means of systems of differential equations e.g., the Krebs cycle [18], the amino acid biosynthetic pathways [19], the oxidative phosphorylation subsystem [20], the glycolytic subsystem [21], the transduction in G-protein enzyme cascade [22], the gene expression [23], the cell cycle [24]. Likewise, in order to understand the emerging dynamics in a multienzymatic set dissipatively structured we have also researched the yeast glycolytic subsystem by using a system of differential equations with delay [25]. In these studies we have analyzed different attractor dynamics linked to Hopf bifurcations [26]–[28], tangent bifurcations [29], the classical period-doubling cascade preceding chaos [30], persistent behaviors [31]–[33] and the multiplicity of coexisting attractors in the phase space [28], [30].

In all these studies, it is assumed that each metabolic subsystem forms a unique dynamical system [9]. The subsystems carry out their activity with autonomy between them and play distinctive and essential roles in the cell [34].

Therefore, a dissipative metabolic network can be considered as a super-complex dynamic structure which integrates a set of different dynamic systems (the metabolic subsystems) forming a dynamical-super-system [10].

The first model of a DMN was developed in 1999 [11] which allowed for the observation of a singular Systemic Metabolic Structure, characterized by a set of different metabolic subsystems always locked into active states (metabolic core) while the rest of the dissipative catalytic subsystems presented on-off dynamics. In this first numerical work it was also suggested that the Systemic Metabolic Structure could be an intrinsic characteristic of metabolism, common to all living cellular organisms [11], [12].

Afterward, in 2004 [35] and 2005 [16], several studies implementing flux balance analysis in experimental data supported new evidence of this Systemic Functional Structure. Specifically, a set of metabolic reactions belonging to different anabolic processes which remain active under all investigated growth conditions was observed. The rest of the enzymatic reactions belonging to different pathways remain only intermittently active. These global catalytic processes were verified by *Escherichia coli*, *Helicobacter pylori*, and *Saccharomyces cerevisiae* [16], [35].

Extensive analyses for DMNs have shown that the Systemic Functional Structure is very robust and stable [36]. Moreover, it has been observed that this global dynamic structure shapes a unique dynamical system, in which self-regulation and long-term memory properties emerge [15]. Long-term correlations have been observed in different experimental studies, e.g., in the quantification of DNA patchiness [37], in physiological time series [38], [39], in NADPH series [40], in DNA sequences [41], [42], in K+ channel activity [43], mitochondrial processes [44] and neural electrical activity [45], [46].

Recently, it was possible to quantify the bio-molecular information flows in a single metabolic subsystem [26], and in a DMN in which the emergence of an effective connectivity structure was also observed [14]. This functional structure of biomolecular information flows is modular and the dynamical changes between the modules correspond to metabolic switches which allow for critical transitions in the metabolic subsystem activities. According to these results [14], the Systemic Metabolic Structure is not only characterized by a metabolic core and self-organized multienzymatic complexes in an on-off changing state but also it shapes a sophisticated structure of effective information flows which provides integrative coordination and synchronization between all the metabolic subsystems [10], [14].

Understanding the systemic mechanisms involved in the regulation of the cellular enzymatic activities in the complex conditions prevailing inside the cell is a key issue for the contemporary biological thought. Here, in order to research the systemic processes involved in the regulation of the catalytic activities, the emerging biochemical data have been analysed using statistical mechanics tools; in concrete, by employing the use of a Boltzmann machine we have built a Hopfield network representing the dynamical properties of the DMN.

The results show that the multienzymatic activities of the network are governed by systemic attractors in which the stored catalytic information patterns can be correctly recovered from specific input stimuli.

The systemic attractors regulate all catalytic network patterns, modify the efficiency in the connection between the multyenzymatic complexes, and stably retain these modifications.

The dissipative metabolic network can behave as an attractor metabolic network which exhibits associative memory properties.

## Model and Methods

### 1. Dissipative Metabolic Networks

Dissipative metabolic networks (DMNs) are dynamical systems basically formed by a given number of interconnected metabolic subsystems (MSbs) which represent self-organized multienzymatic complexes [10], [12].

The metabolic subsystems are interconnected by biochemical substrate fluxes and three classes of regulatory signals: activatory (positive allosteric modulation), inhibitory (negative allosteric modulation) and all-or-nothing type (which correspond to the regulatory enzymes of covalent modulation), for more details see [14].

Each subsystem transforms the input substrate fluxes and regulatory signals into the output catalytic activity. The input-output conversion is performed in two stages. In the first one, the input fluxes are transformed in an internal enzymatic activity of the subsystem by means of flux integration functions. In the second stage, the received regulatory signals modify the internal enzymatic activity converting it into output catalytic activity.

The flux integration functions are based in the quantitative catalytic studies of the amplitude and frequency of the glycolytic patterns obtained by Goldbetter and Lefever in [47], [48] under dissipative conditions.

Each allosteric signal has a 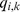 regulatory coefficient which represents the influence of the different activatory and inhibitory modulators on the metabolic subsystems. The index i in 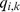 denotes the subsystem that is regulated, and the index k denotes the element (amplitude, baseline or frequency, as will be defined in the next subsection) of the i-th subsystem that is regulated.

If the signal is of all-or nothing type, then it will use a parameter δ which is called the threshold value (the level of the enzymatic covalent regulatory activity). When a given threshold value is reached, it fully inhibits the activity of the MSb.

In agreement with experimental observations, the output activity of all the enzymatic subsystems may be oscillatory or steady state and comprise a very large number of distinct activity regimes [9], [10]. When a subsystem shows an activity with rhythmic behaviour the output catalytic activities present nonlinear oscillations with different levels of complexity, as it could be expected in cellular conditions. Therefore, in the subsystems a large number of transitions between periodic oscillations and steady-states including deterministic chaotic patterns may emerge [15].

### 2. Subsystem Activities

All the explicit details on how DMNs are constructed can be found in [11], [12] and here they are sketched in what follows.

Formally, we assume that the activity of the i-th enzymatic subsystem is defined by
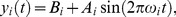
(1)where 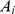 is the amplitude of oscillation, 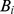 is the baseline and 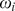 is the oscillation frequency. Moreover, to have 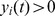 we assume that 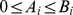 and the baselines and frequencies are bounded values, so there exists 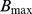 and 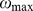 such that 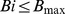 and 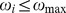 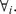


In this way, the activity of each subsystem 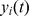 is characterized by three variables 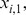 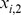 and 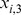, with values between 0 and 1 such that
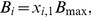
(2)

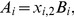
(3)

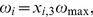
(4)


A subsystem is inactive when 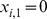, and is in a steady state when 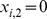 or 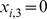.

We fix 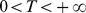 as the total duration of the process and 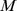 as the number of transitions, and define 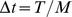 as the time interval during which the oscillations is maintained in the m-th time interval between 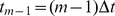 and 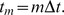 In that interval, the activity of the i-th subsystem is determined by the vector 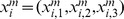 and the state matrix by
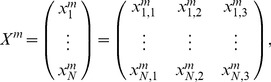
(5)which characterizes the whole DMN system, with *N* the total number of subsystems.

### 3. Flux Integration

Let us suppose that the i-th subsystem receives a flux from the j-th. Its internal activity represented by 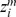 will be computed by three flux integration functions:
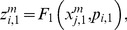
(6)

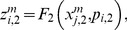
(7)

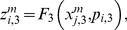
(8)Where 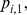 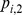 and 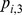
are parameters associated to the flux integration function which are characteristic of each metabolic subsystem, and the 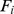 are piecewise linear approximations for nonlinear functions obtained by Goldbeter and Lefever in [48]. One way to reproduce the shape of the functions reported in [48] is to take:
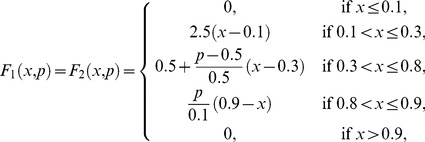
(9)and



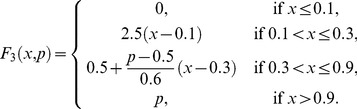
(10)When a subsystem receives different fluxes from at least two subsystems, we compute the arithmetic mean of the F-values previously calculated.

### 4. Regulatory Signal Integration

In this second stage, the internal activity values are modified using the signal integration functions, which depend on the combination of the received regulatory signals and their corresponding parameters (regulatory coefficients). In the metabolic subsystems, the existence of some regulatory enzymes (both allosteric and covalent modulation) increases the interconnection among them. The allosteric enzymes present different sensitivities to the effectors, which can generate diverse changes on the kinetic parameters and in their molecular structure; likewise, the enzymatic activity of covalent modulation also presents different levels of regulation. These effects on the catalytic activities are represented in the dynamical system by the regulatory coefficients and consequently each signal has an associated coefficient which defines the intensity of its influence.

There exist three kinds of signal integration functions:

Activation function AC.
Inhibition function IN.
Total inhibition function TI.

In this way, to compute 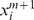 from 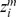 the i-th subsystem receives enzymatic regulatory signals from r subsystems and they work sequentially computing.
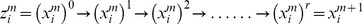
(11)where each step depends on the signal type. From
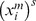
to 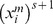 if the signal is AC and is received from the j-th MSb
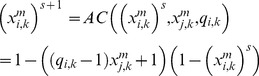
(12)for k = 1, 2, 3 and 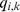 are regulatory coefficient to each allosteric activity signal which represents the sensitivity to the allosteric effectors.

If the allosteric signal is inhibitory
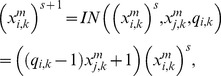
(13)and, finally, if the signal is of the total inhibition type
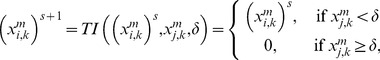
(14)where δ, the threshold value, is the regulatory coefficient associated to each enzymatic activity signal of covalent modulation which defines the intensity of its influence.

### 5. Metabolic Network Generation

First, we have fixed the following elements as control parameters: (1) 18 subsystems in the DMN, (2) up to three substrate input fluxes for each subsystem (each MSb can receive a maximum of three substrate fluxes and it is not restricted the number of flows leaving of them), (3) three input regulatory signals for each metabolic subsystem and (4) the same number of signals per class (allosteric activation, allosteric inhibition and covalent modulation). Certain metabolic subsystems may receive a substrate flux from the exterior and we have arbitrarily fixed the MSb3 and the MSb10 for this function.

Having fixed these elements, the structure of the network has been randomly configured, including: (1) the topology of flux interconnections and regulatory signals, (2) the 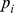 parameters associated to the flux integration functions, (3) the 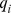 regulatory coefficients to the allosteric activities, and (4) the values of the initial conditions in the activities of all metabolic subsystems (Table in Supporting Information S3).

The values of 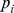 and 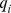 are random numbers between 0 and 1. The changes in the parameters 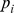
modify the flux integration functions. The values of 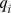 represents the influence level of the allosteric regulatory signals (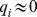 for a low level and 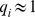 for a high level). The random values of the parameters 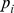 and 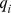 originate metabolic networks with a great variety of catalytic activities in each subsystem.

We have taken the constants 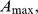 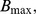 and 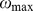 equal to 2, and δ = 0.54 the threshold value of the regulatory coefficient associated with the covalent modulation signal which defines the intensity of its influence.

Finally, given *T* and *M* we calculate the activity matrices 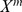 for *m* = 1,…, *M* using the flux integration functions and regulatory signals.

After numerical integration of the selected network, we generate a discrete time-series for the 3-tuples 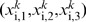. For all cases, the series of baseline, amplitude and frequency are analyzed after 1000 transitions.

### 6. Representation of the Activity of the Metabolic Subsystems

We consider a number *N* of transitions. At the *k-th* iteration-step we suppose that the oscillation is harmonic, Eqs. (1–4). The duration of the harmonic oscillation is a given parameter 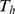 independent on the stage and on the subsystem. Along the two stages, a mixed transition regime is maintained with a duration 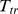
which is independent of the stage number and of the subsystem. If the transition goes from the *k-th* stage to the *(k+1)-th* stage then, during the 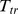 seconds of the transition regime, the activity is given by a function of the form 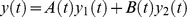, where 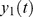 is the activity corresponding to the prolongation in time of the previous harmonic activity in the *k-th* stage, and 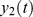 is the back-propagation in time of the subsequent harmonic activity in the *(k+1)-th* stage. The numbers 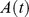
and 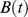 are time dependent and indicate the weights with which the activities of the subsystem in the previous and posterior stage are present during the transition time. At the beginning of the transition, say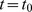, 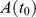 is 1 and 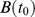 is 0, and at the end of the transition, say 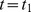, 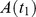 is 0 and 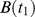 is 1. At the rest of the transition times 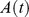 and 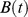 vary according to 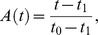 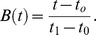 Finally, during the transition time the activity is given by
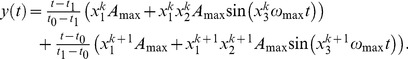
(15)


The transition regimes are combinations of two harmonic oscillations with nonconstant coefficients 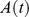 and 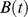 depending on time. Thus, the introduction of these transition regimes provokes the emergence of nonlinear oscillatory behaviors, both simple and complex.

### 7. Brief Justification on the Use of the Boltzmann-Gibbs Distribution in DMNs

We have shown that the enzymatic activity for each MSb in the DMN can be very complex and highly non-linear; however, to understand the global-emerging properties within the DMN is not necessary to incorporate all the details in each MSb. This general flavor, it is well-known in Physics, and in particular the Statistical Mechanics is the discipline for pooling the dynamics of all the (many) system constituents (ie. *the micro-states*) to come out with the time-evolution of a few global measures, the ones which can properly characterize the system as a whole (ie. *the macro-states*).

In a general sense, the approach of going from micro to macro states is not straightforward and to make it possible, one needs first to define the probability distribution of having a macroscopic state based on a microstates configuration. By using this distribution, one can connect micro to macro-states by properly averaging over microstates configurations. In particular, here we will make use of the widely-known Boltzmann-Gibbs distribution [49]–[51], i.e.,
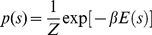
(16)in which 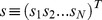 is the microstates vector, 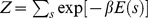 the partition function (i.e. a normalization factor), 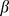 the Boltzmann constant and 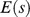 the energy associated to the configuration 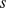.

### 8. The Utilization of Binary Variables in the Modeling of Enzymatic Activity and its Similarity to the Modeling of Neural Networks

A DMN is as a network of 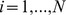 different MSbs, each one manisfesting a highly nonlinear dynamics. To make possible its description through Eq. (16), we need first to determine the possible states for the microscopic variables. Here on, we will represent the enzymatic activity for each subsystem by a binary variable, with states 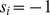 for inactivation and 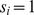 for activation. Although this assumption can seem too simple to describe the real dynamics, however, this is not necessary the case; for instance, it is well understood from neural networks studies, where the input-output relation for individual neurons is well-known to be highly non-linear and noisy (due to the stochastic nature of the neural activity) that binary states can work well to understand global net properties; thus ignoring most of the intrinsic details, the two-states neuronal dynamics can be sufficient for capturing some of the relations between macroscopic variables and neuron dynamics (cite for instance, [49]–[51]).

**Figure 1:**
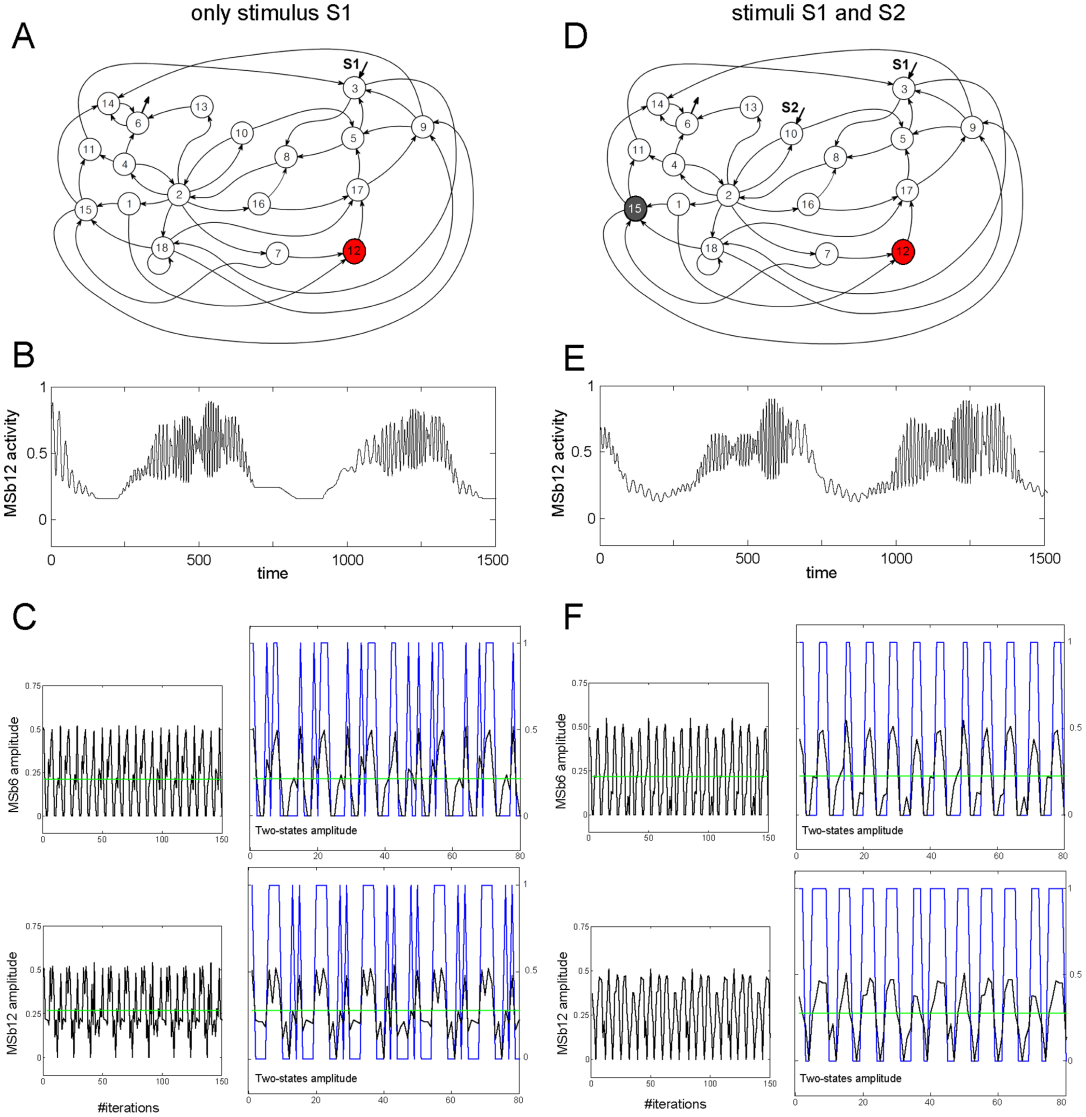
Dynamic catalytic behavior in the dissipative metabolic network (DMN). A,D: metabolic network formed by 18 self-organized multienzymatic complexes (metabolic subsystems); it is shown the interconnection by substrate fluxes and the substrate input fluxes. For simplification in the illustration, the topological architecture of the regulatory signals affecting the DMN is shown in Fig. S1. The network was studied in two different external conditions: condition I, in which only the stationary stimulus S1 was affecting the DMN (left column in panel) and condition II, with two substrate input fluxes S1 and S2 (right column). A: A systemic metabolic structure spontaneously emerges in the network in which the enzymatic subsystem MSb12 is always active (i.e. the metabolic core, red circle) whereas the rest of enzymatic subsystems exhibit on-off changing states (white circles). D: In the condition II the network preserves the metabolic core (red circle) but the MSb15 becomes in a permanent off-state (black circle). B: for condition I, an example of the enzymatic activity of the MSb12 (metabolic core) which presents different catalytic transitions between periodic oscillations and steady states, and (E) same as in B but for condition II. C,F: Time series of the amplitude of several catalytic activity oscillations as a function of the iterations number. Green lines represent the average value of the amplitude in the whole time series. In blue we are plotting the two-states representation of the amplitude time series, 1 for values higher than the green line and 0 for lower values. This figure was slightly adapted from Fig. 2 in (De la Fuente et al. 2011).

The enzymatic activity of the MSbs is represented by a continuous signal; thus, the two-states time-series of activity is achieved simply by looking at each time-instant when the activity is upper or below a threshold of one half the maximum value achieved along the total time-series (cf. the two-states time-series colored in blue in Figure 1).

### 9. The Connection between the Boltzmann-Gibbs Distribution and the Dynamics of Neural Networks

An important reason for the success in the understanding of the collective properties in neural networks in the last decades was the possibility of having the Boltzmann-Gibbs distribution coinciding with the stationary distribution of the neural network dynamics [49]–[52]. This allowed applying some of the Statistical Mechanics techniques to make the mapping between microstates configurations and macroscopic properties. In particular, of special interest was the use of the mean field methods, which based on the Boltzmann-Gibbs distribution, allowed for the first analytical solution to the retrieval properties and storage in the so-popular Hopfield nets; the model [52] and its solution [53].

In concrete, four different reasons made possible to study the neural network dynamics by using the Boltzmann-Gibbs distribution: 1) the neuronal activity was represented by binary variables, 2) the synaptic connections (or weights) were considered to be symmetric, 3) the input-output relation in the activity of each neuron was described by a sigmoid function, 4) the updating for the neural activity was sequential, the well-known *Glauber dynamics* [49]–[52].

During Glauber dynamics one neuron is selected at random at each time-instant and after it is updated with a probability which depends on its current state, its neural threshold, the total input arriving to that neuron and the temperature parameter, which is simply a parameter accounting for random (“not-coming from the input”) fluctuations in the neural activity.

Under these four considerations, the stationary probability distribution resulting from the detailed-balance condition is the Boltzmann-Gibbs distribution. If 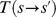 is the transition probability in the movement 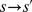, detailed balance ensures that the constraint 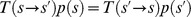 holds for any 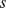 and 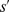. That is, Glauber dynamics (i.e. sequential updating) defines a specific choice for the transition probability to move from configuration 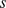 to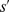, see for instance [49], which allows to have the following stationary probability:
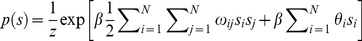
(17)in which 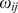 represents the weight from neuron 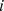 to 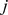 and 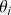 the firing-threshold for neuron 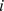. The Botlzmann constant here introduces the temperature parameter, 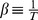.

A simple comparison between Eq. (16) and Eq. (17) gives that
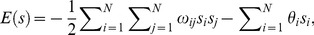
(18)which is the Energy function governing the neural network dynamics, and by analogy, in this paper modeling the enzymatic activity in the DMN.

### 10. The Use of the Boltzmann Machine to Learn the Boltzmann-Gibbs Distribution

The aim of the Boltzmann Machine is to learn from the metabolic data the 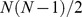 different values of 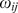 (a symmetric matrix with all elements in the principal diagonal equal to zero) and the
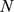
values 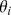 to then use Eq. (17) to obtain global properties of the system by averaging over the Boltzmann-Gibbs distribution.

It is important to remark that the purpose of this paper is not to give a detailed derivation of the algorithm for the Boltzmann Machine; in contrast, we only present it in its simpler formulation, but further details can be found in [54], [55]. At time zero, the parameters 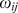 (weights) and 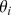 (thresholds) are randomly initialized. As in any learning process, some of the data is used for *training* the learning algorithm. At each iteration, weights and thresholds are updated with a value which is proportional to the difference between the statistics computed directly from the data (the one used for training) and the statistics performed by the Boltzmann-Gibbs distribution with the new updated parameters 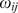 and 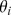. This procedure is repeated until algorithm convergence. In such a way, the Boltzmann machine after convergence ensures that the statistics achieved through the *learned* Boltzmann-Gibbs distribution (hereon, the model) coincide with the one directly computed from the data (more concrete, both model and data have the same average activity and pair-wise correlations).

We provide here a mathematical formulation for the previous explanation. After random initialization of weights and thresholds the specific iterative algorithm is given by:
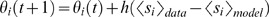
(19)

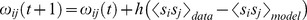
(20)with 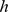 sufficiently small to ensure convergence and where expectations are given by



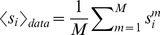
(21)

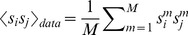
(22)

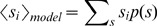
(23)

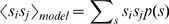
(24)


In each iteration, the probability 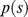 in Eqs. (23) and (24) is the one given by Eq. (17) with the new values 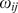 and 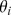 resulting from Eqs. (19) and (20). The index 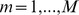 is denoting the different data points which are chosen for algorithm training.

Note that the sums appearing in the expectations given by Eqs. (23) and (24) are involving 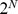 different terms (the number of all possible microstates), and for large networks this number becomes computationally intractable. A common strategy in Statistical Mechanics is the use of Monte Carlo methods, but these methods, although in principle might provide exact calculations, they become very slow for large networks. Alternatively, one can use approximate methods as the achieved by the mean field approximation (details below).

### 11. A Simplified (but Very Efficient) Learning Strategy in Boltzmann Machines with no Hidden Units

Although the learning algorithm given by Eqs. (19) and (20) together with the use of Monte Carlo methods to compute the expectations in Eqs. (23) and (24) is exact [54], [55], for large networks, the algorithm convergence with the use of Monte Carlo methods can be very slow, and other approximations have to be done (for a comparison of methods see [56]).

In particular, we will assume here that all neurons are susceptible for learning (so there are not any hidden units, see [54], [55]). And in this case, the learning in the Boltzmann Machine can be simplified substantially. In particular, we will apply here an efficient and fast method which was elegantly developed by Kappen and Rodriguez [57]. In concrete, Kappen and Rodriguez added better corrections to the solely mean field assumption by applying results from the linear response theory. In this situation, and for the case of not hidden units, the iterative learning algorithm required by Eqs. (19) and (20) has a unique (stable) fixed-point (*fp*) solution which is given by:
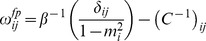
(25)

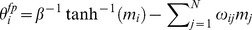
(26)where 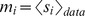 is the mean value in the data and 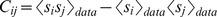 the correlations, cf. Eqs. (21) and (22).

Notice that to obtain Eqs. (25) and (26) one has to assume that the neuronal dynamics is stochastic and represented by the probability for a neuron to fire, i.e.
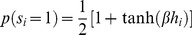
(27)the so-called Glauber dynamics [49]–[51]. Here, 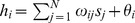 refers to the *local field* arriving to neuron *i*. One can see that (as it occurs for any probability) Eq. (27) satisfies that 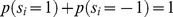.

Notice that Eqs. (25) and (26) have the term 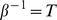 on the right-hand side in contrast with Kappen and Rodriguez in [57] in which they fixed 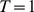 arguing that the value of the temperature can finally be rescaled in both the weights and thresholds. This assumption is true, but however, we have preferred to preserve the temperature parameter in our derivation of Eqs. (25) and (26) to have the possibility of studying the temperature effects in the learning procedure; thus, in the high temperature regime (low 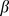) will make the term 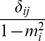 to be dominant versus 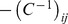 in the calculation of the weights; similarly, 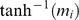 will dominate versus 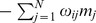 for the threshold calculation. Thus, high temperature values will ignore network effects, ie. contributions 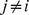 into 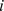, and only the terms with no interactions will prevail at high temperature.

### 12. Two Examples on the Use of the Boltzmann-Gibbs Distribution for Calculation of Network Properties: Shannon Entropy and Average Energy under the Mean Field Approximation

Known the Boltzmann-Gibbs distribution given by Eqs. (16) and (18) is possible to compute different macroscopic variables representing some of the global net properties. Thus, for instance, one can compute the net Shannon Entropy, which defined as
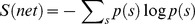
(28)is accounting for the average uncertainty in the network activity, which in the case of base-2 logarithms is given in bits, it accounts for the amount of information which is on-average required to describe the dynamics of the DMN [58]. Notice that this information is directly computed from the time-series of enzymatic activity.

Another important connection between Shannon entropy is its relation with the Mutual Information, a measure for the amount of uncertainty reduction in one variable by knowing another. It can be easily proven that the Mutual information of one variable with itself is the Shannon Entropy. Thus, this self-information has been interpreted as a measure of stored information in the time-series.

Another net observable one can measure having the Boltzmann-Gibbs distribution is the mean Energy, which defined a
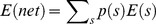
(29)it can account for the network stability; the smaller the mean Energy the more stable is the net configuration.

For the calculation of both Entropy and Energy given by Eqs. (28) and (29), one needs to evaluate the total number of possible microstates, equal to 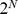 with N the network size. We have mentioned before that an alternative to that is the use of the mean field approximation, similar to what has already widely used for the modeling of neural networks, for details [49]–[51]. In this paper, we have computed both 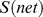 and 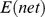 by using the mean field (*mf*) approximation, which is a particular situation in which the probability given by Eq. (17) has the following factorial form
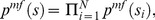
(30)with



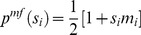
(31)Notice that it is satisfied that 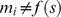; in concrete, the expectation 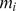 is given by the mean field solution [49]–[51], ie.
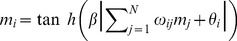
(32)


Thus, using Eqs. (30–32), Eqs. (28) and (29) become
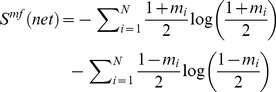
(33)and
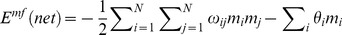
(34)


### 13. Reading-out Hopfield Memories from the Attractor States in DMNs

Eqs. (25) and (26) are the result of the learning algorithm given by Eqs. (19) and (20) in absence of hidden units, under the mean field approximation in addition with some corrections based on the linear response theory, details in [57]. Here, we explore the possibility of reading-out Hopfield memories from the *learned* weights given by Eq. (25).

More concrete, we have assumed that the learned weights given by Eq. (25) are the result of a Hebbian rule similar to the one considered in Hopfield nets (see [52], and [49]–[51] as well). Although this problem (given the weights to determine the set of previously encoded memories) is ill-posed as there exists an infinite number of possible memories to be stored consistent with the same weights matrix, here we will show a specific procedure which allows the finding of a set of Hopfield memories which are locally stable and controls the DMN dynamics versus other memories which are locally unstable.

In the following lines, we detail this method. Firstly, we consider the most general (and simple) Hebbian rule, i.e.
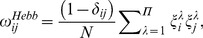
(35)where 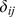 is the Kronecker delta operator and 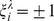 represent the value of the 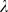 memory at site 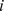. Eq. (35) is the result of encoding-and-storing 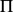 different memories in the weights matrix.

Networks of binary neurons connected by weights given by Eq. (35) have been widely popular, the so-called Hopfield nets [1]–[4]. Interestingly, these networks manifest associative memory, which means that the different attractors in the system dynamics are corresponding to one of the different stored memories 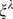. This case, named of *pure attractors,* is the most simple situation but it is also possible to have *mixed* states, in which the attractors correspond to a combination of some of the different encoded memories.

Next, we assume that weights given by Eq. (35) can be represented by the ones learned from the data, Eq. (25). In concrete, our criteria to search the different stored memories 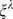 is to minimize the Mean Square Error; that is, to find the *best memories* which minimize the cost given by
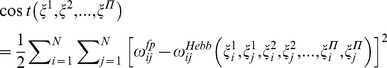
(36)with 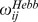 given by Eq. (35).

Several concerns have to be made before performing the minimization of the cost given by Eq. (36): 1) there are 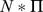 *unknown variables* (the best memories) and 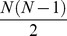 *observations* (given by the matrix 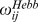 which is symmetric and with diagonal-terms equal to zero). Therefore, the system is overdetermined for 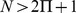. 2) For binary memories, the number of possible states in the search-space is 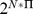, which indicates that the optimization is in principle hard. 3) the cost minimization has to be done over variables which are discrete, implying that standard analytical methods existing for optimization (which make use of the derivatives of the cost function) such as the Newton method, Conjugated Gradient, or Gradient Descent are not applicable to this problem (further details for these methods see [59], [60]). For discrete optimization, other methods have to be applied; among others, they are very popular Dynamic Programming or Heuristic Methods (see [61] and references therein). Herein, we have performed for 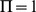 an exhaustive search among all possible memories and compute the minimum cost for each of the memories, i.e., after an exhaustive search we have provided the Least Squares Error (LSE) solution. For 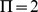 a genetic algorithm has been used to minimize the cost given by Eq. (36), details below.

The solution to Eq. (36) constitutes the essential of our method that can be summarized as the following: from time-series data of enzymatic activity, the matrix 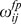 is obtained using Eq. (25). Then, we test the possibility of such weights resulting from a storing-encoding rule of 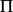 memories, like the one in Eq. (35). The best memories, according to the LSE criteria, are the one that makes 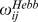 to be most similar to 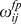.

### 14. Validating the Associative Memory in the DMN; or How much Locally Stable are the Metabolic Memories

To test the validity of the solutions obtained after minimization of the cost given by Eq. (36), we have simulated the dynamics of a Hopfield network with weights given by Eq. (25), thresholds given by Eq. (26), encoding rule given by Eq. (35), and metabolic memories corresponding to the ones minimizing the cost given by Eq. (36). Hereon, we will refer to this as the Hopfield net which is equivalent to the DMN.

If the network manifests associative memory, the activity patterns given by the metabolic memories must correspond with local minima of the dynamics. This can be easily addressed by studying how far the net activity goes after perturbing it when initially was fixed to the activity of one of the metabolic memories. The time evolution of the net activity is computed in Monte Carlo Steps (MCS), one MCS corresponding to N different movements per site in the net activity [49]–[51]. Around a local minimum, fluctuations can push the net activity outward the attractor, but after a transitory the net activity converges back to the attractor. For initial conditions which are not local minima, fluctuations rapidly provoke the net activity to scape permanently from the attractor.

The distance from the initial activity configuration to the evolving net activity has been defined as 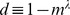, with 
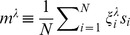
 the usual overlap measure, a Hamming distance between the net activity and the 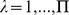 encoded memory. Further details in [49]–[51].

In the Hopfield net simulations, fluctuations are coming from the temperature parameter, 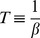 in Eq. (17), which introduces random fluctuations in the input-output relation for each subsystem. In such a way, those fluctuations provide a self-mechanism to perturb the initial condition of the net activity.

### 15. The Use of a Genetic Algorithm to Approximate the Least Squares Error Solution in Discrete-optimization

In the search of the 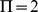 binary metabolic memories, there are more than 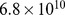 possibilities to be explored for the cost evaluation, cf. Eq. (36). An exhaustive method in the search (as the one performed for the case 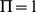) is computationally hard, so we alternatively have approached a genetic algorithm, a heuristic-optimization technique which, inspired in the process of natural evolution, is mixing and mutating different solutions in order to get a minimize the fitness function given by Eq. (36). Here, we briefly explain its roots, but for further details see for instance [62].

Initially, a genetic algorithm defines a set (“*population”*) of candidate solutions (“*individuals”*) for the specific problem in question. The cost associated to each solution is ranked between the worst and the best solution, in a cost-function basis. Next, in an iterative fashion some of the good solutions (*the parents*) are probabilistically selected according to their cost, then mixed (using a *crossover* operator) and changed (via a *mutation* operator) to generate new solutions (*the childs*) that will replace the high-cost ones from the previous iteration; thus, imitating the evolutionary principle of *survival of the fittest* (i.e. the one with least cost).

As the genetic algorithm solutions are based on a random search, it do exists the possibility that the genetic algorithm becomes trapped in a local minimum; to avoid that possibility the method usually is executed several times and the best solution is selected.

In this work, we use a population of 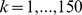 individuals, each one corresponding to one possible set of 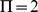 metabolic memories, i.e., memories A and B represented by 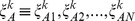 and 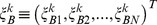. Thus, an individual is formed by two vectors of size N. The population is evaluated using the Eq. (36) and sorted with a cost-basis criteria. The 10% of the best solutions are passed through the next generation (so they are new candidates to be chosen); the 90% of the left are modified using crossover and mutation.

Crossover operation works as follows: first, two pairs of individuals are randomly selected, and the best two of the four are considered to be crossed (named *dad* and *mum* respectively). After chosen the integer vector-position i at random, the new crossed individual is built taken the dad positions from the 1st to the ith and the mum positions from the (i+1)th to the Nth. Technically speaking it is said that there is a tournament selection with a tournament size equal to 2. Notice that this crossover is performed in the two 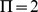 memories A and B.

Mutation selects at random one individual (a two N-size vectors) and one integer vector position and flips its value. The mutation operator is applied several times.

Finally, the new individuals are in-cost-evaluated and the procedure is repeated for a given number of iterations.

Notice that the main purpose of the use of the genetic algorithm was not to perform a fine optimization of the fitness function given by Eq. (36). For that reason, we have not explicitly studied.

## Results

To research the systemic biomolecular mechanisms involved in the regulation of the enzymatic activity when several enzymatic sets interact with each other we have built a Dissipative Metabolic Network (DMN) with 18 metabolic subsystems, each one representing a self-organized multienzymatic complex; hereon, we will name indifferently catalytic subsystem, multienzymatic subsystem or MSb.

The DMN has been built according to the following concerns:

The number of catalytic subsystems in the DMN is fixed to 18.
The maximum number of substrate input fluxes for each subsystem is fixed to 3.
The number of input regulatory signals for each metabolic subsystem is fixed to 3.
There are three classes of regulatory signals in the network: activatory (positive allosteric modulation), inhibitory (negative allosteric modulation) and all-or-nothing type (which correspond to the regulatory enzymes of covalent modulation). The regulatory signals are affecting all the catalytic subsystems and it is not required any flux relationship.
Every metabolic subsystem receives both fluxes and regulatory signals.
It exists a balanced number between allosteric activation, allosteric inhibition and regulatory signals of covalent modulation.

The network architecture was built by choosing a random topology of substrate flux interconnections and regulatory signals. Moreover, we have randomly defined the parameters describing the flux-integration functions, the regulatory coefficients of the allostetic activities and the values of the initial conditions for all metabolic subsystems (Tables in Supporting Information S2 and Supporting Information S3).

Dissipative metabolic networks are open systems and some of the metabolic subsystems may receive a substrate flux from the exterior. Here, we have arbitrarily fixed the MSb3 and MSb10 to receive external stimuli; in concrete we have applied constant substrate inputs of S1 = 0.54 to MSb3 and S2 = 0.16 to MSb10.

Figure 1A, 1D illustrates the organization of substrate fluxes and input fluxes of the DMN. Notice that the MSb18 is presenting a self-catalytic process. The complex architecture of the regulatory signals affecting the network is depicted in Figure S1.

### 1. Catalytic Dynamics of the Self-organized Multi-enzymatic Complexes Under Systemic Conditions

We have studied first the catalytic dynamical patterns emerging in the network when only a stationary input flux of substrate S1 is considered (hereon Condition I).

Under this external condition, a systemic metabolic structure emerges spontaneously in the network: the MSb12 is always in an active state (i.e. metabolic core), whereas the rest of multi-enzymatic subsystems exhibit intermittently activity transitions between on and off states.

All metabolic subsystems present complex output patterns with large transitions between different oscillatory behaviors (350 transitions per period). Figures 1B,1E show a representative time series of the catalytic activities belonging to the MSb12 where only 30 transitions between oscillatory behaviors are depicted.

In another condition, the biochemical network was receiving two external and simultaneous stimuli S1 and S2 (hereon Condition II).

Under this new external perturbation the same network undergoes a drastic reorganization of its catalytic dynamics showing flux plasticity which involve persistent changes in the enzymatic activities of all subsystems (Figure 1B vs 1C, and 1E vs 1F), and structural plasticity which, in this case, implies a persistent change in the state of the MSb15 i.e., in condition I the MSb15 was locked to an on-off changing dynamics and in condition II the MSb15 exhibits a permanent off-state (Figure 1A,1D).

In condition II all subsystems change their catalytic patterns presenting complex activity with 105 transitions (per period) between oscillatory and steady state behaviors (in Figure 1E an example of only 30 transitions in the catalytic activity of the MSb12 can be observed). Notice that despite the non-linear behavior of the time-series, its dynamics is purely deterministic and noise-free. Despite the drastic catalytic changes observed in the time evolution of the dynamics of the all subsystems, the network preserves its Systemic Metabolic Structure, i.e., the MSb12 is the metabolic core (which is always active) and the rest of active subsystems continue exhibiting on-off intermittently dynamics.

The complex dynamic behaviors which spontaneously emerge in the network have their origin in the regulatory structure of the feedback loops, and in the nonlinearity of the constitutive equations of the system. Therefore, the mechanism that determines the complex catalytic behaviors is not prefixed in any particular location of the metabolic system and there are no specifically designed rules that force the system to present these complex transitions in the output catalytic activities for the all metabolic subsystems.

Once the main dynamical behaviors of the dissipative metabolic network have been reported, we have used the amplitude of the catalytic patterns (see some examples in Figures 1C, 1F ) to study in a quantitative way, some macroscopic properties of the network using statistical mechanic tools. In concrete by exploiting a Boltzmann machine algorithm we have described the stationary properties of the DMN by a Boltzmann-Gibbs distribution; allowing studying some network properties, such as the energy, the Shannon Entropy and the mapping of the local minima of the dynamics with Hopfield-like attractors, reporting in such a way on the possibility of that the DMN manifests associative memory. Details below.

### 2. Reading-out Boltzmann-Gibbs Distributions from Attractors in DMNs by Using a Boltzmann Machine

We have applied a Boltzmann machine to learn from time-series of enzymatic activity the matrix of weights connectivity and the vector of thresholds; cf. Eqs. (25) and (26). The Boltzmann machine was applied to the two stimulation conditions (explained before). Results for condition I are given in Tables 1 and 2; for condition II results are given in Tables 3 and 4.

**Table 1:** Matrix of weights connectivity: condition I (only stimulus S1).

0.00	1.09	4.25	−1.57	1.30	−0.36	−1.86	0.79	−1.48	−2.43	−0.02	0.09	1.97	0.58	0.00	−0.70	−2.93	0.47
1.09	0.00	−6.12	2.82	−4.29	1.79	3.55	−3.68	−0.60	0.23	−1.46	3.07	−1.29	0.76	0.00	0.25	1.86	1.22
4.25	−6.12	0.00	4.15	−6.66	0.24	7.41	−3.17	8.79	18.31	−3.19	6.53	−4.42	−0.06	0.00	−3.65	10.13	−0.65
−1.57	2.82	4.15	0.00	2.53	−0.18	−3.67	3.32	−0.67	−1.00	1.02	−2.81	−0.97	−0.63	0.00	0.16	−1.14	1.06
1.30	−4.29	−6.66	2.53	0.00	0.93	3.71	−2.89	1.48	−0.60	−2.03	5.42	−1.44	2.49	0.00	1.48	−0.32	2.39
−0.36	1.79	0.24	−0.18	0.93	0.00	−0.42	−0.54	2.64	−3.85	−0.65	1.97	3.00	1.41	0.00	1.82	−0.51	0.03
−1.86	3.55	7.41	−3.67	3.71	−0.42	0.00	5.02	−2.17	−1.33	1.15	−1.80	1.65	−0.36	0.00	−0.45	−2.74	0.94
0.79	−3.68	−3.17	3.32	−2.89	−0.54	5.02	0.00	−0.40	−2.43	−3.00	2.69	1.35	0.55	0.00	2.55	−0.18	0.34
−1.48	−0.60	8.79	−0.67	1.48	2.64	−2.17	−0.40	0.00	−0.78	−0.35	−1.20	0.31	−0.09	0.00	−0.16	−1.56	0.92
−2.43	0.23	18.31	−1.00	−0.60	−3.85	−1.33	−2.43	−0.78	0.00	−1.63	−0.39	5.63	2.34	0.00	6.18	−5.95	2.49
−0.02	−1.46	−3.19	1.02	−2.03	−0.65	1.15	−3.00	−0.35	−1.63	0.00	1.74	2.92	0.92	0.00	1.75	−0.50	0.16
0.09	3.07	6.53	−2.81	5.42	1.97	−1.80	2.69	−1.20	−0.39	1.74	0.00	0.05	−2.43	0.00	0.34	1.28	−0.76
1.97	−1.29	−4.42	−0.97	−1.44	3.00	1.65	1.35	0.31	5.63	2.92	0.05	0.00	−0.49	0.00	−2.18	3.66	0.28
0.58	0.76	−0.06	−0.63	2.49	1.41	−0.36	0.55	−0.09	2.34	0.92	−2.43	−0.49	0.00	0.00	−0.90	0.82	−0.67
0.00	0.00	0.00	0.00	0.00	0.00	0.00	0.00	0.00	0.00	0.00	0.00	0.00	0.00	0.00	0.00	0.00	0.00
−0.70	0.25	−3.65	0.16	1.48	1.82	−0.45	2.55	−0.16	6.18	1.75	0.34	−2.18	−0.90	0.00	0.00	3.06	−2.00
−2.93	1.86	10.13	−1.14	−0.32	−0.51	−2.74	−0.18	−1.56	−5.95	−0.50	1.28	3.66	0.82	0.00	3.06	0.00	1.61
0.47	1.22	−0.65	1.06	2.39	0.03	0.94	0.34	0.92	2.49	0.16	−0.76	0.28	−0.67	0.00	−2.00	1.61	0.00

Each cell in the table corresponds with a given weight; ith row, jth column is correponding to 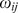. Notice that the matrix is symmetric and with the principal diagonal equal to zero. Mean val 2.71; std dev 34.36; min val −150.11; max val 151.23.

**Table 2:** Vector of thersholds: condition I (only stimulus S1).

48.31	7.31	15.89	−8.89	−4.70	−3.29	81.05	3.09	−33.86	29.12	−0.29	−74.57	6.77	−11.91	−33.86	13.93	−71.88	48.34

Each cell for each threshold value. Mean val 0.59; std dev 39.04; min val −74.57; max val 81.05.

**Table 3:** Matrix of weights connectivity: condition II (both stimuli S1 and S2).

0.00	1.09	4.25	−1.57	1.30	−0.36	−1.86	0.79	−1.48	−2.43	−0.02	0.09	1.97	0.58	0.00	−0.70	−2.93	0.47
1.09	0.00	−6.12	2.82	−4.29	1.79	3.55	−3.68	−0.60	0.23	−1.46	3.07	−1.29	0.76	0.00	0.25	1.86	1.22
4.25	−6.12	0.00	4.15	−6.66	0.24	7.41	−3.17	8.79	18.31	−3.19	6.53	−4.42	−0.06	0.00	−3.65	10.13	−0.65
−1.57	2.82	4.15	0.00	2.53	−0.18	−3.67	3.32	−0.67	−1.00	1.02	−2.81	−0.97	−0.63	0.00	0.16	−1.14	1.06
1.30	−4.29	−6.66	2.53	0.00	0.93	3.71	−2.89	1.48	−0.60	−2.03	5.42	−1.44	2.49	0.00	1.48	−0.32	2.39
−0.36	1.79	0.24	−0.18	0.93	0.00	−0.42	−0.54	2.64	−3.85	−0.65	1.97	3.00	1.41	0.00	1.82	−0.51	0.03
−1.86	3.55	7.41	−3.67	3.71	−0.42	0.00	5.02	−2.17	−1.33	1.15	−1.80	1.65	−0.36	0.00	−0.45	−2.74	0.94
0.79	−3.68	−3.17	3.32	−2.89	−0.54	5.02	0.00	−0.40	−2.43	−3.00	2.69	1.35	0.55	0.00	2.55	−0.18	0.34
−1.48	−0.60	8.79	−0.67	1.48	2.64	−2.17	−0.40	0.00	−0.78	−0.35	−1.20	0.31	−0.09	0.00	−0.16	−1.56	0.92
−2.43	0.23	18.31	−1.00	−0.60	−3.85	−1.33	−2.43	−0.78	0.00	−1.63	−0.39	5.63	2.34	0.00	6.18	−5.95	2.49
−0.02	−1.46	−3.19	1.02	−2.03	−0.65	1.15	−3.00	−0.35	−1.63	0.00	1.74	2.92	0.92	0.00	1.75	−0.50	0.16
0.09	3.07	6.53	−2.81	5.42	1.97	−1.80	2.69	−1.20	−0.39	1.74	0.00	0.05	−2.43	0.00	0.34	1.28	−0.76
1.97	−1.29	−4.42	−0.97	−1.44	3.00	1.65	1.35	0.31	5.63	2.92	0.05	0.00	−0.49	0.00	−2.18	3.66	0.28
0.58	0.76	−0.06	−0.63	2.49	1.41	−0.36	0.55	−0.09	2.34	0.92	−2.43	−0.49	0.00	0.00	−0.90	0.82	−0.67
0.00	0.00	0.00	0.00	0.00	0.00	0.00	0.00	0.00	0.00	0.00	0.00	0.00	0.00	0.00	0.00	0.00	0.00
−0.70	0.25	−3.65	0.16	1.48	1.82	−0.45	2.55	−0.16	6.18	1.75	0.34	−2.18	−0.90	0.00	0.00	3.06	−2.00
−2.93	1.86	10.13	−1.14	−0.32	−0.51	−2.74	−0.18	−1.56	−5.95	−0.50	1.28	3.66	0.82	0.00	3.06	0.00	1.61
0.47	1.22	−0.65	1.06	2.39	0.03	0.94	0.34	0.92	2.49	0.16	−0.76	0.28	−0.67	0.00	−2.00	1.61	0.00

Similar to Table 1, ith row, jth column is corresponding to 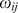. Notice that the matrix is symmetric and with the principal diagonal equal to zero. Mean val 0.37; std dev 2.83; min val −6.66; max val 18.31.

**Table 4:** Vector of thersholds: condition II (both stimuli S1 and S2).

−4.38	−3.53	−1.52	−0.20	−5.05	−2.61	−0.26	−4.69	−3.08	−7.33	−6.45	6.16	5.99	3.23	NaN	3.52	−4.56	3.34

Each cell for each threshold value. Mean val −1.26; std dev 4.29; min val −7.33; max val 6.16.

Note: The NaN number in position 15 is because the MSb15 is in an off-state, which is equivalent to have a positive infinite threshold. This value has been removed and not considered in the calculation of both mean and standard deviation.

The weights data given in Tables 1 and 3 have a standard deviation which is higher than the one in standard Hopfield nets. This occurred in both stimulation conditions I (mean = 2.71, std dev = 34.36) and II (mean = 0.37, std dev = 2.8). In standard Hopfield nets, weights are given by Eq. (35) and typically are generated by using the so-called orthogonal memories, in which their probability distribution is assumed to be factorisable, i.e. 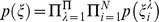 with 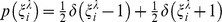. For this case, it is straightforward to proof that the weights generated through Eq, (35) have a mean value of zero and a standard deviation of 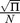, so for large size nets the standard deviation is practically zero.

**Figure 2:**
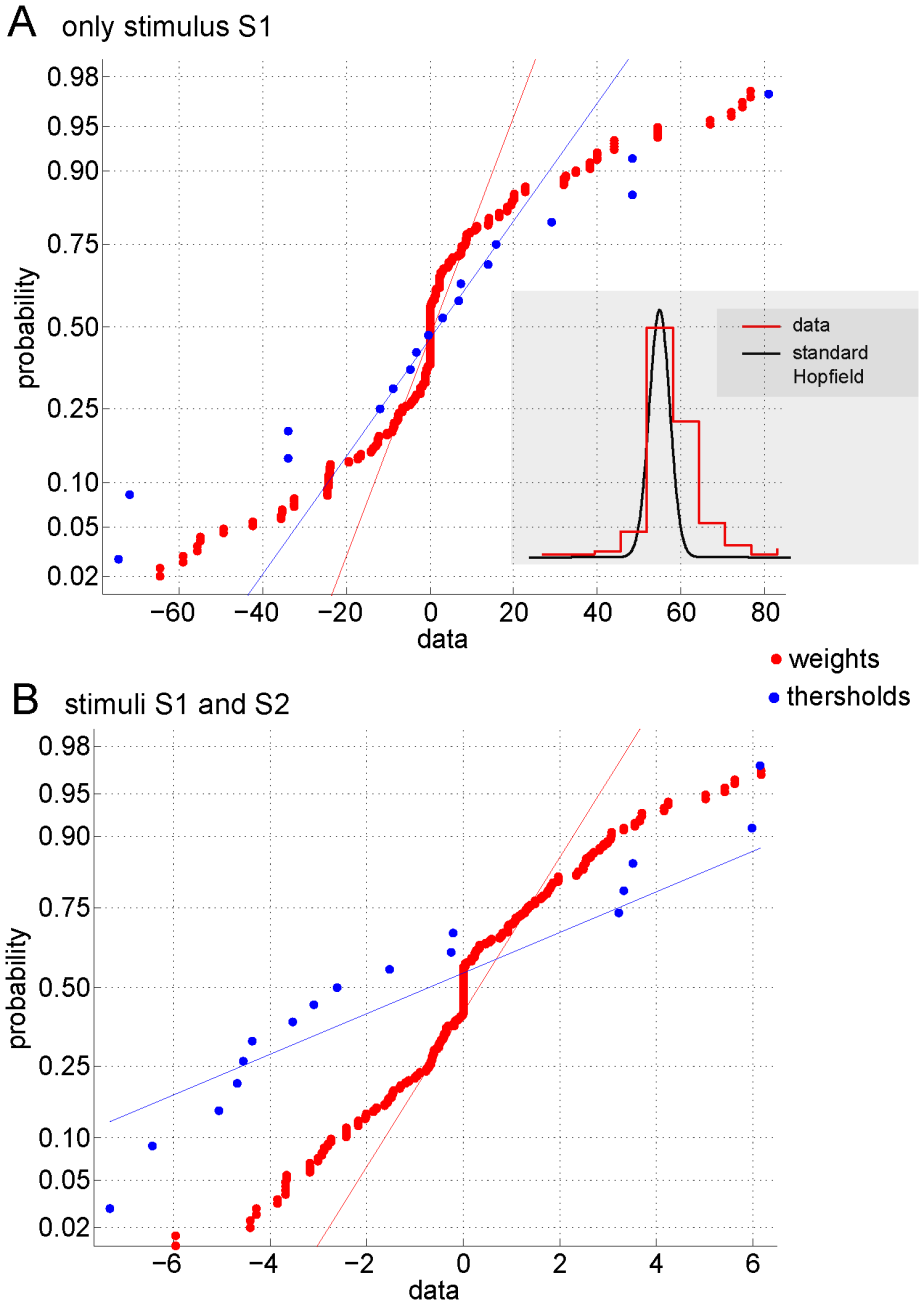
Evidence for non-Gaussianity in weights and thresholds obtained through the Boltzmann machine. Normal probability plot; data deviations from the Gaussian distribution are graphically mapping to the deviations from the straight line (built with a purely Gaussian distribution). A: stimulation condition I in which only the stimulus S1 is presented. B: condition II: both stimuli S1 and S2 are applied to the DMN. For both conditions, weights (colored in red) and thresholds (in blue) are strongly non-Gaussian. This is important as standard Hopfield nets assume that weights are following a Gaussian distribution with mean zero and standard deviation 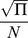; this is represented in the inset of panel A with a black line for 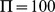.

The reason for this discrepancy comes from the fact that the weights in the DMN data are strongly non-Gaussian. This is illustrated in Figure 2, weights colored in red, thresholds in blue. The normal probability plot is a graphical statistical test to validate how much Gaussian is the data. Data purely Gaussian had been depicted precisely on the straight line (the more non-Gaussian is the data, the bigger deviations appear from the straight line).

In most of the situations Hopfield networks assume weights which are Gaussian distributed (black line in the inset in Figure 2A). However, the constraints for the thresholds are more flexible, and different considerations have been assumed before: Gaussian thresholds, all thresholds equal to a constant, a different constant for each threshold, oscillatory thresholds, etc. For details see [49]–[51].

**Figure 3:**
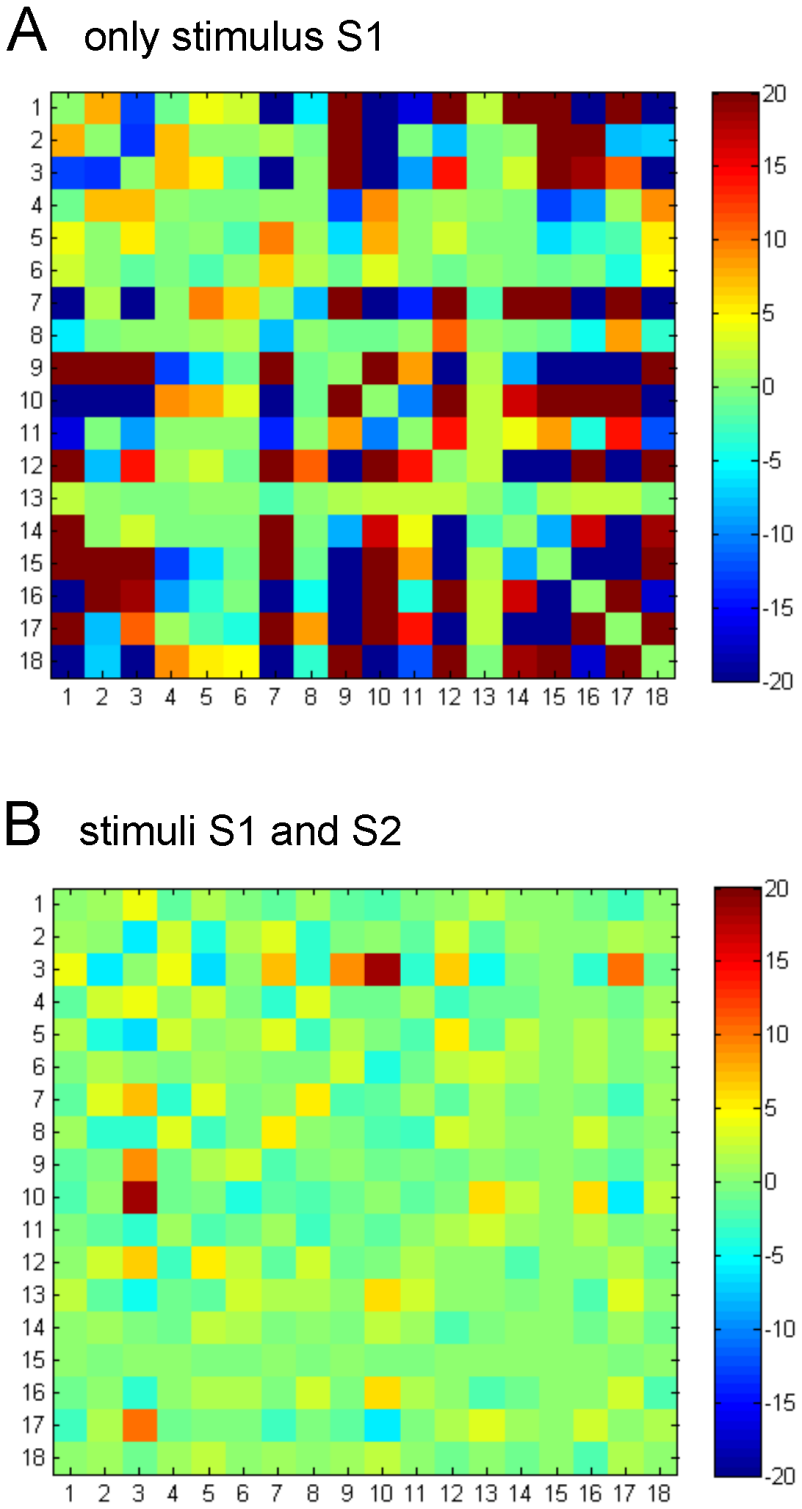
Weights connectivity matrix learned from the DMN by the Boltzmann machine. For the two stimulation conditions, we plot the two matrices of weights connectivity that are the result of the learning by the Boltzmann machine. Notice that although the mean values in both matrices are small (2.71 in panel A and 0.37 in panel B), the variance in panel A is much higher compared to the one in panel B (A: 34.36, B: 2.83). The tables with these values and their corresponding statistics are given in Tables 1 and 3.

For both stimulation conditions I and II, weights have both positive and negative values, meaning that the activity belonging to two interacting subsystems (say 1 and 2) can either be positively correlated (when one is up the other is up) and anti-correlated (when one is up the other is down). This is illustrated in Figure 3. For the two stimulation conditions, we plot the two matrices of weights connectivity, result from Eq. (25). Notice that although the mean values in both matrices are small (2.71 in panel A and 0.37 in panel B), the variance in panel A is much higher compared to the one in panel B (A: 34.36, B: 2.83). Thus, from Figure 3 it is observed how the learned weights clearly depend on the different stimulation condition.

The results for the thresholds are given in Tables 2 and 4. In general, one can see that, similar to the weights, thresholds have positive and negative values; the more positive is the threshold value, the less excitable is the associated MSb.

### 3. Shannon Entropy as a Measure for the Information in the DMN

After convergence in the learning, the Boltzmann machine allows to obtain a weights matrix and a vector of thresholds such as the stationary probability of the dynamics of the DMN coincides with the Botlzmann-Gibbs distribution with an Energy function given by Eq.(18). This is very important as it allows describing global (stationary) properties of the DMN from the dynamics of the catalytic subsystems. The Shannon Entropy given by Eq. (28) can be interpreted as the information in bits that can be read-out from the time series describing the dynamics of the DMN. To do this, we have considered Eqs. (30) and (31), the so-called “mean field approximation”. Under these conditions, the Shannon Entropy is given by Eq. (33).

We have computed the Shannon Entropy for the two stimulation conditions I and II. It had a value of 10.45 for condition I and 10.56 for condition II. Thus, the Shannon Entropy did change very little (with a small relative error) in the two stimulation conditions. Notice that, in contrast with the Energy in Eq. (34), the Entropy given by Eq. (33) does not depend on weights and thresholds; thus, the Entropy is a measure which has not a direct dependence on the temperature, as all the temperature dependencies come from the weights and the thresholds cf. Eqs. (25) and (26).

It is important to remark that we have computed the Entropy for all enzymatic subsystems except the number 15. The reason was that for condition II, the MSb 15 was in the off state, so giving a not-a-number (NAN) contribution to the Shannon Entropy, c.f. Table 4. For comparison purposes, we have skipped as well the contribution of the MSb 15 in the condition I. Thus, the stored information was about 10.5 for 17 subsystems in total, which gives about 0.61 bits of information per each subsystem.

The fact that the Shannon Entropy kept almost unchanged and independently on the stimulation condition, it can give cues about a possible invariant in the information stored in the dynamics of the DMN.

### 4. Net Energy as a Measure of the Global Stability in the DMN

The use of the Botlzmann machine allows for describing different stationary properties at the network level for the DMN by exploiting the statistics of the Boltzmann-Gibbs distribution. The net energy (see methods) is a convenient measure for global stability of the dissipative metabolic network. Similar to the calculation of the Shannon Entropy (corresponding to the metabolic network information), we have assumed also for the energy calculation the mean field approximation. The energy in this case is given by Eq. (14).

We have computed the net energy for the two conditions I and II. Network energy had a value of −158.34 for condition I versus −27.83 for condition II, thus when only the stimulus S1 is stimulating the biochemical system, the DMN is 5.69 times more stable than when the two stimuli S1 and S2 are activating the network. This occurred for a temperature value of T = 0.5. The effect of the temperature was not very relevant though; thus for T = 0.1 we obtained −158.79 (condition I) and −28.13 and for T = 1.0, −157.78 (I) and −27.47 (II). Thus, increasing the temperature, the Energy function also increased, making the network to be less stable. However, despite of this (well-known) tendency, the DMN is about 6 times more stable in condition I in comparison with condition II, and this fact was independent on the value of the temperature.

Interpreting in terms of the energy the transition of the MSb15 from an on-off state in condition I to an off state in condition II, it says that the dynamical condition in which all subsystems except the metabolic core are in on-off states is about 6 times more stable in comparison with the situation in which the MSb 15 was in the off-state. In other words, the situation in which all non-core subsystems are oscillating is energetically 6 times cheaper than the one to force the MSb15 to be in the off-state.

### 5. Testing Attractors Stability of the Metabolic Memories

**Figure 4:**
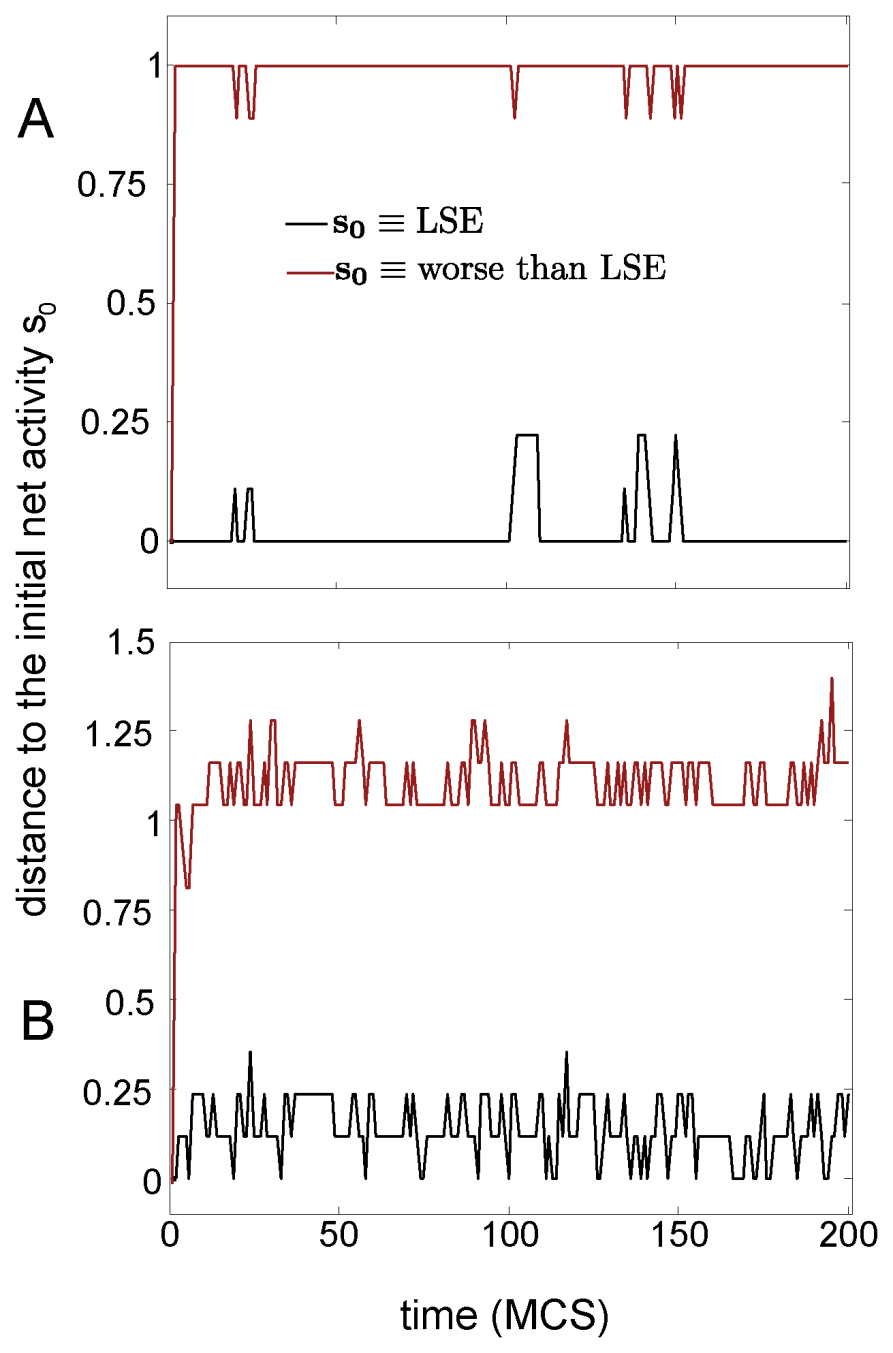
The metabolic memories are local minima of the DMN dynamics (case 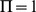). For only 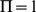 metabolic memory encoded in the weights, we plotted the time evolution of the distance between the evolving net activity and the initial net activity 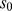, fixed to be equal to the activity of the metabolic memory (details in methods). A,B: Time is given in units of Monte Carlo Steps (MCS). Black lines correspond to fixing the initial activity to the memory provided by the LSE solution. Red lines correspond to fixing the activity to an arbitrary memory that has a higher cost than the LSE (i.e. worse than LSE). A: stimulation condition I (only stimulus S1). B: stimulation condition II (both stimuli S1 and S2). In this case of 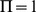 the LSE solution has been found by using an exhaustive method in the search-space and providing the LSE memory (details in the text). Fluctuations in the time-series are originated by a temperature parameter of T = 0.7.

We have tested the attractors stability by looking into the dynamics of the Hopfield net which is equivalent to the DMN (see methods). By fixing the initial net activity to the activity of one metabolic memory, one can study how much stable is that memory by looking the time evolution of the net activity. This is represented in Figure 4. Black lines represent the evolving net activity when the initial condition was equal to the memory provided by the Least Square Error (LSE); another arbitrary memory with a higher cost than the LSE is plotted with red lines (i.e. worse than LSE). Figure 4 shows that for both stimulation conditions I and II, the LSE metabolic memory is a local minima as the net activity random-fluctuates around it, but without escaping from it; this does not occur, however, with the memory having a higher cost than the LSE, from which after very few time-iterations the network activity goes far from the initial state, and never returns back to the attractor, thus indicating that the memory is locally unstable. In such a way, the metabolic memory provided by the LSE solution is a local minimum of the equivalent Hopfield dynamics of the DMN, an evidence for associative memory.

**Figure 5:**
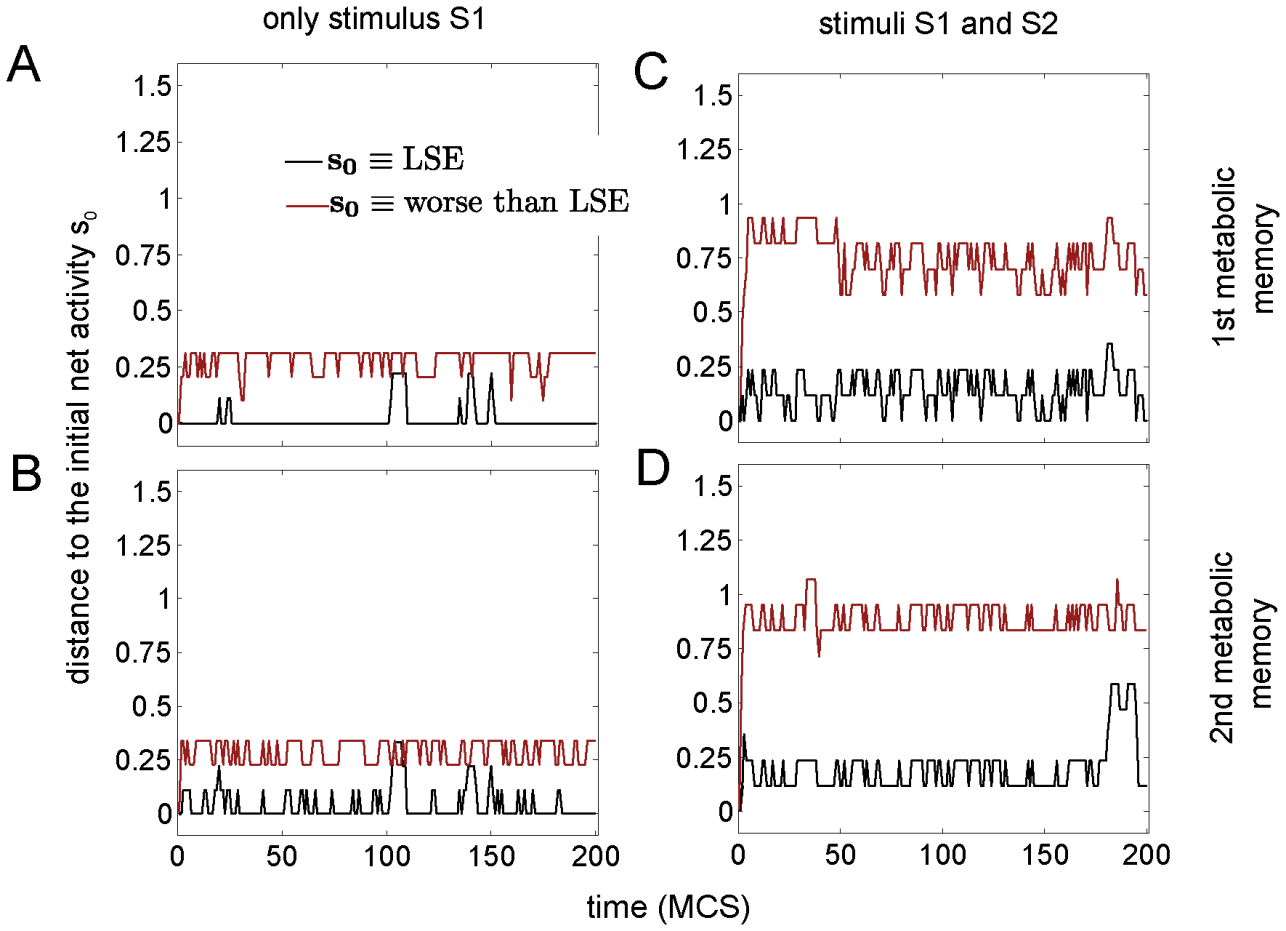
The metabolic memories are local minima of the DMN dynamics (case 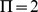). This figure is similar than Fig. 3 but now there are 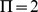 metabolic memory encoded in the weights. A,C: the first metabolic memory in both cases LSE and worse than LSE conditions. B,D: similar than in A,C but for the second metabolic memory. A,B: stimulation condition I (only stimulus S1). C,D: condition II (both stimuli S1 and S2). In this case of 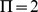, the LSE solution has been found by using an genetic algorithm for minimization of the cost given by Eq. (36), details in the text. The temperature parameter is fixed to T = 0.7.

For 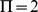 metabolic memories the cost minimization has been performed by using a genetic algorithm (see methods). Results are plotted in Figure 5. The time-evolution of the net activity dynamics is represented for the two stimulation conditions I and II with different initial conditions: for the first metabolic memory, this is depicted in Figures 5A and 5C. For the second memory, in 5B and 5D. Similar to the case of Figure 4, the two metabolic memories provided by the LSE are local minima of the dynamics (black lines) but this does not happen for another arbitrary solution with a higher cost than the LSE (red lines). Thus, indicating that the equivalent Hopfield net of the DMN can manifest associative memory also for 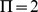.

## Discussion

One of the most important goals of contemporary biology is to understand the elemental principles and quantitative laws governing the enzymatic processes in cellular conditions.

Here, in order to research the systemic regulatory mechanisms involved in the control of the cellular enzymatic activity we have quantified macroscopic properties and essential dynamic aspects of a dissipative metabolic network formed by 18 metabolic subsystems each one representing a set of enzymes functionally associated and dissipatively structured.

The enzymes, proteins and RNAs characterized by exhibiting catalytic activity, are the main functional molecules of cell, and they do not function in isolation of one another [4] but by shaping self-organized multienzymatic complexes called metabolic subsystems which exhibit autonomous oscillatory and quasi-stationary activity patterns [9], [10]. These multienzymatic subsystems are responsible for almost all the biomolecular transformations, which, globally considered, are called cellular metabolism, and they represent the nodes of the dissipative metabolic networks.

In the multienzymatic network that we have analyzed, the metabolic subsystems are interconnected through a complex topological structure of biochemical signals formed by substrate fluxes and three classes of molecular regulatory processes: activatory (positive allosteric modulation), inhibitory (negative allosteric modulation) and an all-or-nothing type (which corresponds to the regulatory enzymes of covalent modulation). These kinds of biochemical signals constitute the main mechanisms of the enzymatic interconnection in living cells [13], and they may be understood as metabolic synapses i.e., the functional connection processes among self-organized multienzymatic complexes through which biomolecular information flows from one metabolic subsystem to another [14].

The preliminary analysis of the network catalytic activities shows that the enzymatic patterns spontaneously self-organize themselves, leading to the emergence of a Systemic Metabolic Structure characterized by a set of different enzymatic reactions which are always locked into active states (metabolic core) while the rest of the catalytic processes are only intermittently active. This global dynamic structure is in agreement with experimental results [16], [17], [35].

Moreover, our study shows how complex catalytic patterns emerge in the subsystems, which exhibit hundreds of different transitions between oscillatory behaviors, and some steady states, as it is expected on the cellular conditions [9], [10].

It is interesting to remark that the dissipative metabolic network that we have studied self-adjusts the internal enzymatic activities to the external environmental changes by means of metabolic flux plasticity (changes in the physiological values of the metabolic synapses which lead to a differential catalytic activity of subsystems) and metabolic structural plasticity (persistent change in the state of the multienzymatic subsystems which can be in an active state, in an *on-off* changing state or an inactive state: all these catalytic states are also due to the metabolic synaptic changes). The global self-regulations of the enzymatic processes by means of metabolic flux plasticity and structural plasticity have been experimentally observed in several unicellular organisms as the main systemic metabolic mechanisms for the adaptation to external perturbations [17], [35].

As a first preliminary conclusion of our work, the dissipative metabolic network analyzed here, despite its remarkable conceptual simplicity, shows fundamental organizational elements which are present in all unicellular metabolic organisms, i. e., (1) the enzymes functionally associated forming self-organized multienzymatic complexes, (2) the main biochemical mechanisms for the metabolic synapses (substrate fluxes, allosteric processes, and regulatory mechanisms of covalent modulations), (3) the emergence of dissipative catalytic patterns, (oscillatory and stationary enzymatic activities in far from equilibrium conditions), (4) the global enzymatic self-organization (presence of the Systemic Metabolic Structure which is characterized by an enzymatic core and intermittently active catalytic processes), and (5) the systemic enzymatic self-regulation (adaptation to environmental changes by means of flux plasticity and structural plasticity).

### Consequences of the Use of the Boltzmann Machine in the DMN: Lyapunov Function, Energy Landscape, Hopfield-like Attractors and Metabolic Associative Memory

The main conclusions of the use of the Boltzmann machine are the following:

- The catalytic dynamics of the dissipative metabolic network are dependent on the Lyapunov function i.e, the energy function of the biochemical system, which is a fundamental element in the regulation of all the enzymatic activities.

Started in any initial system state after an external stimulus, and due to the global multienzymatic dynamics, the metabolic network evolves trying to reduce the energy function, which in absence of noise will decrease in a monotone way until achieving a final system state that is a local minimum of the Lyapunov function.

When the biochemical system state reaches a local minimum, it becomes a (locally) stable state for the enzymatic network.

The dissipative metabolic network has multiple stable states and each one of them corresponds to a specific global enzymatic pattern of the biochemical system.

- The enzymatic activities of the dissipative metabolic network are governed by systemic attractors which are locally stable states, where all the enzymatic dynamics are stabilized.

The minimums of the Lyapunov function correspond to these systemic attractors which regulate the metabolic patterns of the subsystems and determine that the enzymatic network operates as an individual and completely integrated system.

The energy function on which the dynamics of the metabolic network depend has a complex landscape with multiple attractors (local minima).

- The emerging systemic attractors analyzed here are corresponding with Hopfield-like attractors [52] in which metabolic information patterns can be stored.

The biochemical information contained in the attractors behave as metabolic memories i.e., enzymatic activity patterns stored as stable states, which regulate both the permanent changes in the internal catalytic medium as well as the metabolic responses to properly integrate the perturbations coming from the external environment.

When a stimulus pattern is presented to the system, the network states are driven by the intrinsic enzymatic dynamics towards a determinate systemic attractor which corresponds to a memorized biochemical pattern.

The formation of the attractor landscape is achieved by metabolic synaptic modifications in which each biochemical synapse is involved in the storage of the metabolic memories.

Metabolic synaptic plasticity seems to be the basis of the stored memories in the metabolic network.

- A key attribute of the analyzed systemic attractors is that when the dissipative metabolic network (DMN) is stimulated with a pattern input, the enzymatic activities converge to the stored pattern which most closely resembles the input, i. e. the attractors have “associative memory”.

The metabolic network attractors seem to store functional enzymatic patterns which can be correctly recovered from specific input patterns.

Our results explicitly show that indeed, there exists memory attractors and capacity to retrieve binary information patterns (from the weights) which are local minima of the catalytic activity in the DMN.

Thus, if the initial condition of the network dynamics starts within the basin of attraction of such a pattern, the dynamics will converge to the originally stored pattern. As it is well known, this class of “pattern-completion” dynamics has been addressed in Hopfield nets studies for years, and it is generally named “associative memory” [49]–[52].

### Beyond the Mean Field Approximation

We have computed the mean field energy of the equivalent-Hopfield network which is built from the dynamics of the DMN, and we have found some explicit information patterns which are local minima of this energy. Notice that the original dissipative metabolic network can have much more complicated energy landscape than the one approached by the mean field solution. However, even if the calculation of a more exact energy function is beyond the scope of the present work, these results give clues that the local minima of the metabolic net energy can first store information to eventually be retrieved. And this is what researchers in neural networks studies named to have associative memory.

### Encoding and Retrieving Information Patterns: Associative Memory in the DMN

In our case, the synaptic connectivity matrix in the Hopfield network is the result of an adaptation of a Hebbian learning rule (firing together, wiring together) for which “simultaneous activation of two metabolic subsystems leads to pronounced increases in biochemical synaptic strength between both metabolic subsystems”.

The stimuli sequences of vectors which are encoded into the learning rule correspond to an information pattern. In the simplest case, after learning the synaptic weights do not change their strength any more. When connecting metabolic subsystems with the weights matrix, the stable states of the entire network dynamics coincide with one of the stimulus which was stored in the learning rule. Thus, the name of information pattern comes from the fact that the information existing in the weights-encoded vector can be retrieved after learning by simply stimulating the network with a stimulus which is close to the one used for patterns encoding. Therefore, pattern information, encoding and retrieval in the dissipative metabolic network exist in the dissipative metabolic network.

The metabolic network analysed here converts the biomolecular information flows into new activity patterns, modifies the efficiency in the connection between the multyenzymatic complexes, and stably retains these modifications. These enzymatic dynamic behaviors are governed by Hopfield-type attractors, and therefore, the dissipative metabolic network behaves as an attractor metabolic network which has associative memory properties.

Neural networks with associative memory, addressing storage and recollection of encoded patters in the learning rule, have been studied in theoretical neurobiology since longer than 50 years ago. Starting at the preliminary work done by Willshaw et al [63] and continuing by several studies [64]–[66] to finally becoming highly popularized by Hopfield in [52], an article with about twelve thousands citations in Google Scholar.

In these pioneer theoretical studies it was suggested that such associative memory could explain the operational mechanisms for memory in neural systems; recently the theoretical predictions formulated several decades ago have been experimentally validated [67], showing the existence of attractors in neural circuits, thus underlying associative memory.

Here for the first time, we are suggesting that associative memory could be also present in metabolic networks, thus defining the general concept of attractor metabolic network, in which this dynamic behavior, emerging as collective phenomena, might be also observed.

### Shannon Information in the DMN

On the other hand, we have measured the systemic Shannon entropy (under the mean field approximation) of the enzymatic activity in the metabolic network. The Shannon entropy (the average uncertainty in the dynamics of enzymatic activity) is directly related to the concept of information. More precisely, the Shannon entropy coincides with the amount of information which on-average is required to describe the DMN system dynamics [58]. It is important to remark that this information can be naturally read-out from the time-series of enzymatic activity. Interestingly, Shannon entropy is related to the measure of mutual information between two variables as well (the mutual information measures the amount of uncertainty reduction in one variable by knowing another). Concretely, the mutual information of one variable with itself is the Shannon Entropy.

We have computed the Shannon Entropy for the two stimulation conditions: one in which only the stimulus S1 is applied to the metabolic network (condition I), and the other in which both stimuli S1 and S2 are applied (condition II). The Shannon Entropy changed very little in the two stimulation conditions and was about 10.5 bits for 17 multienzymatic subsystems in total, which gives around 0.61 bits per metabolic subsystem.

Both the Energy function and the Shannon Entropy have been computed under the mean field assumption; but other more sophisticated non-equilibrium methods can be approached starting at the Boltzmann-Gibbs distribution, i.e. in Ising-like systems. For a pedagogical book on these methods see [68].

### About the Maximum Capacity for Storing Information Patterns in the DMN

Another important feature of an associative Hopfield network is the maximum number of patterns that the network can be stored to after be retrieved, i.e. the storage capacity. Studies on the storage capacity in Hopfield networks are very abundant as this problem has been widely studied and discussed [49]–[52]. Under many different network assumptions and encoding rules, and beyond specific model details, the storage capacity for recollection memory, which is requiring a full retrieval of the complete encoded memory, it scales with N, the number of nodes. For instance, Hopfield suggested that a network with N nodes could store about 0.15 N patterns in the form of the stable states [52], later, it was theoretically found that the storage capacity is 0.138 N by Amit et al. [53].

Much less studies exist on the storage capacity of Hopfield network regarding familiarity memory [69], [70]; the possibility that the neural network can discriminate previously encoded patterns from patterns which are novel and have not been encoded before. For this “yes or no” discrimination task, the storage capacity has a different nature in the size scaling; it scales with N∧2 versus the N scaling occurring for recollection [70]–[72]. Thus, the same Hopfield network can both perform recollection (full pattern retrieval) and familiarity (the discrimination between one memory which was encoded before or just a novel one, never-seen before), and very interestingly the different memory nature has a very different storage capacity (N∧2 for familiarity vs N for recollection).

### Broad Estimation on the Storage of Metabolic Memories in Prokaryotic Cells

In light of these data, one might wonder how many metabolic information patterns a living cell could possibly store.

At present, the number of metabolic subsystems present in unicellular organisms is unknown, but it is possible to get a rough estimation of its magnitude order.

The genome of E. coli K12, one of the most studied cellular micro-organisms, only contains 4452 open reading frames, of which 2403 (54.1%) have an experimentally determined function, 1425 (32%) were predicted by computational analysis, and the remaining 663 (14.9%) are of unknown function [73]. Total of all, at least 931 correspond to enzymes [74] which are organized into 165 metabolic pathways [75].

However, despite this small number of genes, the amount of all synthesized proteins per cell including the enzymes is very high around 3,600,000 [76] due the existence of multiple molecular copies. A million corresponds to external proteins (flagella and pili), and in the inner part of the cell there is around 2,600,000 (1,000,000 of them are placed in the cytoplasm, excluding 900,000 ribosomal proteins, 80,000 are in the periplasmic area and 100,000 are nucleoid-associated proteins; likewise, the inner membrane contains around 200,000 and the outer membrane exhibits around 300,000 proteins [76]).

Like the rest of cellular organisms, the enzymes and the non-catalytic proteins of the E. coli K12 present a multiplicity of copies which can shape numerous metabolic subsystems, e.g., the number of ribosomes/cell in E. coli range from 6800–72000 units [77] each one of them can be considered a metabolic subsystem [78], the bacterial RNA polymerase holoenzyme responsible for the whole transcription is a complex structure of five subunits which range from 1500–11400 units per cell [79], the glucose-specific permease IICBGlc which form part of multifunctional system for the phosphorylation and translocation of sugar substrates through the cytoplasmic membrane have about 2361 copies in the bacteria [80], the β-galactosidase exhibits 3,000–5,000 molecules per cell when E. coli grown in lactose as the sole carbon source [81], the phosphofructo-kinase possesses about 10483 copies per cell [80]), the phosphoglycerate mutaseA around 6526 enzymes per bacteria [80]), the OmpR molecules about 3037–4628 units per cell [82], etc.

E.coli very possibly exceed 100,000 self-organized multienzymatic subsystems and taking into account this minimal estimation of its magnitude order, the number of functional metabolic memories stored (recollection memory) will be well above 100,000 (the familiarity metabolic memory is even much greater, scaling with N^∧^2), whereas the genetic structural information for this bacteria is around 4,500 genes.

### Broad Estimation on the Storage of Metabolic Memories in Eukaryotic Cells

Eukaryotic cells are larger and more structured than prokaryotic cells, with bigger genomes and much more complex metabolic systems. For instance, a human liver cell only has genetic structural information of 20,000–25,000 genes [83] and exhibits around 13 million ribosomes on the rough endoplasmic reticulum [84].

If we consider only the number of ribosomes as the total number of metabolic subsystems, the minimum number of functional metabolic memories stored (recollection memory) will be well above 13,000,000 in the hepatocyte, and familiarity scaling with N^∧^2.

### Experimental Findings of Biomolecular Information Processing in Unicellular Organisms

Different experimental works have shown that several unicellular organisms possess complex behaviors that are only possible if biomolecular information is stored and processed.

One of the most studied examples of information processing in a single cell is the *Physarum polycephalum*. This unicellular species belongs to the amoebozoa, an important branch of eukaryote evolution, is characterized by a large cytoplasm with many nuclei which remain suspended in a single contiguous protoplasmic volume called plasmodium. The body of the plasmodium contains a network of tubular structures by means of which nutrients and biomolecular elements circulate through the organism in an effective manner [85].

The movement of plasmodium is termed shuttle streaming which is characterized by the rhythmic back-and-forth flow of the biomolecular substances. The dynamically reconfiguring network of tubular structures can redirect the flow of protoplasm towards the plasmatic membrane causing the movement of a mass of flowing pseudopods, and as a result, the organism can crawl over ground at a speed of approximately 1–5 cm/h [86].

The complex protoplasm exhibits a rich spatiotemporal oscillatory behavior, and streams rhythmically synchronised with intracellular oscillatory patterns such as the oscillations in ATP concentration, the plasmagel/plasmasol exchange rhythm and the oscillations in the cytoplasmic free calcium level [87].

In the last several years, a notable number of studies in *Physarum polycephalum* have shown that this cell is able to store information, learn and recall past events.

For instance: this unicellular organism had the ability to find the minimum-length solution between two points in a maze, which has been considered cellular information processing [88], [89] and it has also been verified that the shortest path problem in the maze is a mathematically rigorous solution [90]; the Physarum construct appropriate networks for maximizing nutrient uptakes achieving better network configurations than that of the ones based on the shortest connection of Steiner’s minimum tree [91], [92]; direct experimental studies evidenced that these kind of amoebas can memorize sequences of periodic environmental changes and recall past events during the adaptation of cells to different stimuli (it can even anticipate a previously applied 1 hour cold-dry pattern) [93]; despite being a single multinucleate cell the plasmodium can be used to control autonomous robots [94], [95]; furthermore, it has also been verified that the plasmodium can solve complex multiobjective foraging problems [96]–[98] and other complex problems of optimization by means of adaptive network development [99], [100].

All these experimental results hint at the emergence of an intrinsic memory storage device [100] and a primitive intelligence [88], [93], [101]–[103].

Many other experimental examples show that other different unicellular organisms have sophisticated behaviors including information storage.

A pioneer in revealing such behaviour was H. S. Jennings, who showed many years ago that a single cell such as Paramecium could have a primitive kind of learning and memory [104]. Neutrophils also exhibit a rudimentary memory system in which they are able to ‘‘recall’’ past directions [105]. *Dictyostelium* and *Polysphondylium* amoebae seem to have a rudimentary memory and they can show long directional persistence (∼10 min), being able to remember the last direction that they had just turned [106]. Even individual neurons seem to show short-term memories, and permanent information can be stored when nerve cells in the brain reorganize and strengthen the connections with one another [107].

### Final Summary

We have quantified essential dynamic aspects of a dissipative metabolic network among which we have found that the systemic enzymatic activities are governed by attractors with the capacity to store metabolic information patterns which can be correctly recovered from specific input stimuli (associative memory). As a consequence, the multienzymatic network has the capacity to learn, self-regulate and self-adapt to new external conditions.

In a quantitative manner, our numerical analysis show indications that the systemic catalytic processes of living cells may behave as a functional attractor network and therefore it is endowed of its capacity for storing metabolic patterns.

In light of our results, and, in addition to the relatively small amount of genetic information that characterizes most cells, the metabolic networks of living cells may have far more biomolecular information in the form of functional metabolic memories stored in the connectivity patterns of the self-organized multienzymatic subsystems.

It has not escaped our notice that the possible duality in the storage system of molecular information in cells (structural-genetic and functional-metabolic) are of considerable biological interest.

We are now working on some elementary properties of this dynamic system capable of storing functional metabolic information. Details on the molecular mechanisms that link both storage systems (metabolic memory and genetic memory) will be published later.

Understanding how the enzymes are functionally organized under the complex conditions prevailing inside the cell and which systemic mechanisms are involved in the regulation of the cellular enzymatic activity, are crucial for the unraveling of the fundamental biomolecular dynamics of cellular life.

## Supporting Information

Figure S1 **Sketch of regulatory signals in the DMN.** Each subsystem represents a set of functionally associated enzymes which are dissipatively structured. Three classes of regulatory signals are considered: allosteric activation (blue), allosteric inhibition (red) and covalent modulation (green). Non-directed edges in black represent a superposition of more-than-one classes of signals. For instance, from MSb4 to MSb5 it exist a superposition of the three classes signals.
(TIF)

Supporting Information S1 **Metabolic Subsystems.**
(DOC)

Supporting Information S2 **Parameters of the dissipative metabolic network.**
(DOC)

Supporting Information S3 **Initial conditions of the 18 metabolic subsystems.**
(DOC)
