# Supplementary figures and images for: Attractor Metabolic Networks
Source: PLoS One. 2013 Mar 15;8(3):e58284. doi: 10.1371/journal.pone.0058284 (PMC3598861; doi:10.1371/journal.pone.0058284)

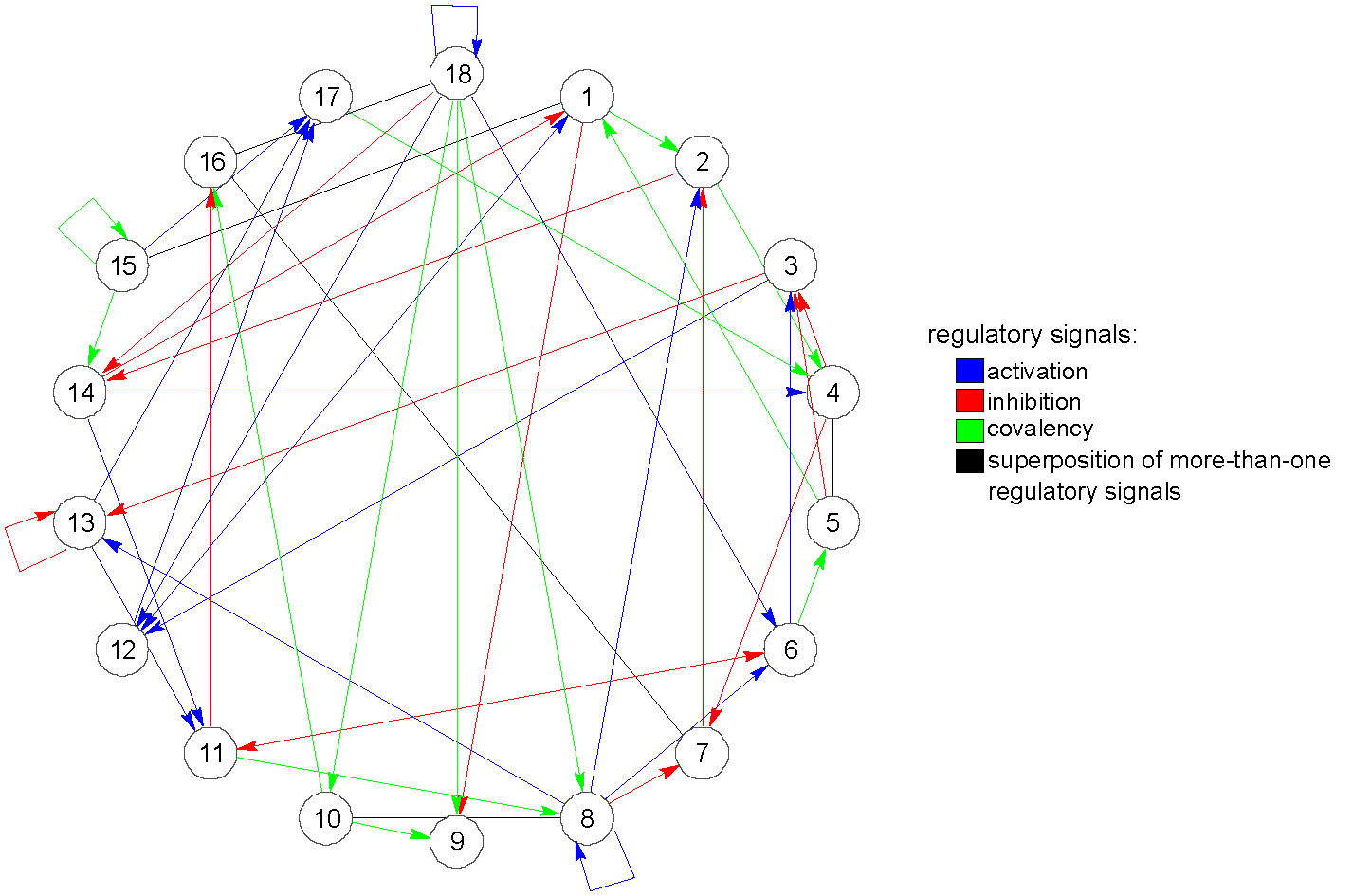

Supplement: Figure S1 — Sketch of regulatory signals in the DMN. Each subsystem represents a set of functionally associated enzymes which are dissipatively structured. Three classes of regulatory signals are considered: allosteric activation (blue), allosteric inhibition (red) and covalent modulation (green). Non-directed edges in black represent a superposition of more-than-one classes of signals. For instance, from MSb4 to MSb5 it exist a superposition of the three classes signals. (TIF) [file pone.0058284.s001.tif]
