# Supplementary material for: Attractor Metabolic Networks
Source: PLoS One. 2013 Mar 15;8(3):e58284. doi: 10.1371/journal.pone.0058284 (PMC3598861; doi:10.1371/journal.pone.0058284)
Supplement: Supporting Information S1 — Metabolic Subsystems. (DOC) [file pone.0058284.s002.doc]

**Supporting Information S1**

**Metabolic Subsystems**

How the enzymes are functionally organized under the complex conditions prevailing inside the cell, and which systemic mechanisms are involved in the regulation of the cellular enzymatic activity, are two crucial issues for the understanding of the fundamental biomolecular dynamics of cellular life.

Most enzymes are proteins, but a few RNA molecules called ribozymes, ribonucleic acid enzyme, also manifest catalytic activity [1,2].

About the first issue, how the enzymes are functionally organized inside the cell, intensive studies of protein-protein interactions have shown that the internal cellular medium is an assembly of supra-molecular protein complexes [3], e.g., the analyses of the proteome of Saccharomyces cerevisiae have shown that at least 83% of all proteins form complexes (containing from two to eighty-three proteins), and their overall enzymatic structure is formed by a modular network of biochemical interactions between multienzyme complexes [4]. This molecular self-organization occurs in all kinds of cells, both eukaryotes and prokaryotes [5-7].

The molecular self-organization of various enzymes in large complexes may allow for the direct transfer of their common intermediate metabolites from the active site of one enzyme to the catalytic centre of the following enzyme without a prior dissociation into the bulk solvent (substrate channelling). This process of non-covalent direct transfer of metabolic intermediates allows for a decrease in the transit time of reaction substrates, originating a faster catalysis through the pathway, preventing the loss of reaction intermediates by diffusion and increasing the efficiency and control of the catalytic processes in the multienzymatic aggregate [8-12]. Substrate channelling may occur within the protein matrix channels or along the electrostatic surface of the enzymes belonging to macromolecular complexes [13-14].

In addition, reversible interactions of multienzyme aggregates with structural proteins and membranes are a common occurrence in eukaryotic cells leading to the emergence of metabolic microcompartments within the soluble phases of cells [15-20].

Prokaryotic cells also exhibit microcompartments, but in this case they have outer shells which are composed of thousands of protein subunits and are filled with enzymes belonging to specific metabolic pathways in the interiors [21-22].

The dynamic biomolecular processes by which microcompartmentation is maintained remains unclear.

The self-organization of cooperating enzymes into multienzyme complexes, the substrate channelling and their integration in microcompartments seem to be central features of cellular metabolism, crucial for the regulation and efficiency of enzymatic processes and fundamental for understanding the molecular enzymatic architecture of cell life.

The self-organization at the enzymatic level presents another relevant characteristic: the emergence of functional structures which allows the temporal organization of catalytic processes.

Apart from forming complex catalytic associations, enzymes can exhibit molecular rhythms which constitute one genuine property of the dissipative self-organization and also allow the functional coordination between different multienzyme complexes [23-25].

In the far-from-equilibrium conditions prevailing inside the cell, the catalytic dynamics of enzymatic sets present transitions between different quasi-stationary and oscillatory molecular patterns.

When the concentration of a particular metabolite does not present oscillating behavior it is usually called steady state. However, in cellular conditions these patterns exhibit slow variations in concentration over time so they are never completely constant. Therefore, the metabolic system will remain in a quasi-steady state when the concentration of a particular metabolite varies over time in a non-oscillating manner [23].

In cells, the proportion between oscillatory and quasi-stationary behaviours is not known but some experimental observations seem to show that the quasi-steady states are much less frequent than the oscillatory processes [26].

During the last four decades, extensive studies of biochemical dynamical behaviors, both in prokaryotic and eukaryotic cells, have revealed that in cellular conditions a spontaneous emergence of molecular oscillations occurs in most of the fundamental metabolic processes. For instance, specific biochemical oscillations (ultradian rhythms) were reported to occur in: free fatty acids [27], NAD(P)H concentration [28], biosynthesis of phospholipids [29], cyclic AMP concentration [30], ATP [31] and other adenine nucleotide levels [32], intracellular glutathione concentration [26], actin polymerization [33], ERK/MAPK metabolism [34], mRNA levels [35], intracellular free amino acid pools [36], cytokinins [37], cyclins [38], transcription of cyclins [39], gene expression [40-43], microtubule polymerization [44], membrane receptor activities [45], membrane potential [46], intracellular pH [47], respiratory metabolism [48], glycolysis [49], intracellular calcium concentration [50], metabolism of carbohydrates [51], beta-oxidation of fatty acids [52], metabolism of mRNA [53], tRNA [54], proteolysis [55] urea cycle [56] Krebs cycle [57] mitochondrial metabolic processes [58] nuclear translocation of the transcription factor [59] amino acid transports [60] peroxidase-oxidase reactions [61] protein kinase activities [62] and photosynthetic reactions [63].

In addition, experimental observations in Saccharomyces cerevisae during continuous culture have shown that practically the majority of metabolome also show oscillatory dynamics [64].

Likewise, it has been observed that genomic activity shows multioscillatory behaviors. For instance, considering only the transcription processes, it has been reported that at least 60% of all gene expression oscillates with an approximate period of 300 min [65]. Other experimental observations have shown that practically the entire transcriptome exhibits low-amplitude oscillatory behavior [66] and this phenomenon has been described as a genomewide oscillation [26,43,64-68].

Evidence that the cells exhibit multi-oscillatory metabolic processes with fractal properties has been reported, and these dynamic behaviors seem to be consistent with scale-free dynamics spanning a wide range of frequencies of at least three orders of magnitude [58].

The temporal organization of the metabolic processes in terms of rhythmic phenomena covers a wide time window with period lengths ranging from milliseconds [69], to seconds [70], minutes [71] and hours [72].

The transition from simple periodic behavior to complex oscillatory phenomena, including bursting (oscillations with one large spike and series of secondary oscillations) [73] and chaos (irregular oscillations), is often observed in metabolic behaviors [74].

From a dynamic point of view, these multienzymatic associations represent dissipative structures in which some functional integrative processes emerge, allowing the reactive coordination between their catalytic parts [23,25].

As a result of the dissipative self-organization, each multienzymatic complex shapes a catalytic entity as whole, where spatial molecular rhythms, temporal molecular oscillations and quasi-steady state patterns can spontaneously emerge.

In addition, the self-organization and the self-assembly processes may allow for reversible interactions between the multienzymatic system and other molecular structures, leading to the formation of a metabolic microcompartment, which provides a discrete reactive space where the intermediate metabolites can be protected from being consumed by competing reactions catalyzed by other multienzyme structures. In the multienzymatic complex, the increase in efficiency and control of the associative catalytic activity can also be improved by other processes such as metabolic channeling [15].

These self-organized multienzymatic complexes associated with other non-catalytic biomolecular structures are called metabolic subsystems [23].

The dissipative metabolic subsystems are thermodynamically advantageous biochemical structures, which act as individual catalytic entities, forming unique, well-defined dynamical systems and their activity is autonomous with respect to the other self-organized multienzymatic subsystems [23,75].

The dissipative mechanisms that provide the spatio-temporal self-organization of the metabolic subsystem activities find their roots in the non-linear regulatory processes which control the dynamics of their irreversible catalytic reactions [76,77].

The non-linearity associated to the irreversible catalytic reactions is the main mechanism which might allow the dissipative metabolic subsystem to work far from the thermodynamic equilibrium [77,78].

Once the irreversible catalytic system operates sufficiently far-from-equilibrium due to the nonlinear nature of its kinetics, the quasi-steady state may become unstable, leading to different dynamical behaviors, and new instabilities can originate the emergence of complex functional patterns [76,77,79].

Dissipative self-organization of multienzymatic complexes allows for the emergence of a rich variety of catalytic patterns and comprises an infinite number of distinct activity regimes [75].

Each metabolic subsystem represents a highly integrated catalytic unit [80], which exhibits the spontaneous self-organization of its enzymatic activity and seems to constitute the fundamental catalytic node of the cellular metabolism networks [80].

The metabolic subsystem seems to be the basic catalytic element for the reactive transformation of cellular metabolic processes, and they are the basis for more complex biomolecular dissipative self-organization at superior structural and functional levels, as for example the Intracellular Energetic Units (ICEU) [81] and the synaptosomes [82].

**Supplementary References:**

1. Cech T (2000) Structural biology. The ribosome is a ribozyme. Science 289(5481): 878–9.

2. Lilley D (2005) Structure, folding and mechanisms of ribozymes. Curr Opin Struct Biol 15 (3): 313–23

3. Pang CN, Krycer JR, Lek A, Wilkins MR (2008) Are protein complexes made of cores, modules and attachments? Proteomics 8 (3): 425–34.

4. Gavin AC, Bosche M, Krause R, Grandi P, Marzioch M, et al. (2002) Functional organization of the yeast proteome by systematic analysis of protein complexes. Nature 415: 141-147.

5. Bobik TA (2006) Polyhedral organelles compartmenting bacterial metabolic processes. Appl. Microbiol. Biotechnol. 70:517–525.

6. Yeates TO, Kerfeld CA, Heinhorst S, Cannon GC, Shively JM (2008) Protein-based organelles in bacteria: Carboxysomes and related microcompartments. Nat. Rev. Microbiol. 6:681–691.

7. Sutter M, Boehringer D, Gutmann S, Günther S, Prangishvili D, et al. (2008) Structural basis of enzyme encapsulation into a bacterial nanocompartment. Nat. Struct. Mol. Biol. 15: 939–947.

8. Clegg JS, Jackson SA, (1990) Glucose metabolism and the channelling of glycolytic intermediates in permeabilized L-929 cells. Arch. Biochem. Biophys. 278: 452–460.

9. Negrutskii BS, Deutscher MP (1991) Channeling of aminoacyl tRNA for protein synthesis in vivo. Proc. Natl. Acad. Sci. USA 88: 4991–4995.

10. Ovadi J, Srere PA (2000) Macromolecular compartmentation and channeling. Int. Rev. Cytol. 192: 255–280.

11. Jorgensen K, Rasmussen AV, Morant M, Nielsen AH, Bjarnholt N, et al. (2005) Metabolon formation and metabolic channeling in the biosynthesis of plant natural products. Curr. Opin. Plant. Biol. 8: 280–291.

12. Jovanović S, Jovanović A, Crawford MR (2007) M-ldh serves as a regulatory subunit of the cytosolic substrate-channelling complex in vivo. J. Mol. Biol. 371: 349–361.

13. Milani M, Pesce A, Bolognesi M, Bocedi A, Ascenzi P (2003) Substrate channeling: Molecular bases. Biochem. Mol. Biol. 31: 228–233.

14. Ishikawa M, Tsuchiya D, Oyama T, Tsunaka Y, Morikawa K (2004) Structural basis for channelling mechanism of a fatty acid bold beta-oxidation multienzyme complex. EMBO J. 23: 2745–2754.

15. Ovádi J, Saks V (2004) On the origin of the ideas of intracellular compartmentation and organized metabolic systems. Mol. Cell. Biochem. 256: 5–12.

16. Monge C, Grichine A, Rostovtseva T, Sackett P, Saks VA (2009) Compartmentation of ATP in cardiomyocytes and mitochondria. Kinetic studies and direct measurements. Biophys. J. 96: 241a.

17. Lunn EJ (2007) Compartmentation in plant metabolism. J. Exp. Bot. 58: 35–47.

18. Saks VA, Monge C, Anmann T, Dzeja P (2007) Integrated and organized cellular energeticsystems: Theories of cell energetics, compartmentation and metabolic channeling. In MolecularSystem Bioenergetics. Energy for Life; Saks, V.A., Ed.; Wiley-VCH: Weinheim, Germany.

19. Monge C, Beraud N, Kuznetsov AV, Rostovtseva T, Sackett D (2008) Regulation of respiration in brain mitochondria and synaptosomes: Restrictions of ADP diffusion in situ, roles of tubulin, and mitochondrial creatine kinase. Mol. Cell. Biochem. 318: 147–165.

20. Saks V, Monge C, Guzun R (2009) Philosophical basis and some historical aspects of systems biology: From hegel to noble - Applications for bioenergetic research. Int. J. Mol. Sci. 10:1161–1192.

21. Yeates TO, Tsai Y, Tanaka S, Sawaya MR, Kerfeld CA (2007) Self-assembly in the carboxysome: A viral capsid-like protein shell in bacterial cells. Biochem. Soc. Trans. 35: 508–511.

22. Fan C, Cheng S, Liu Y, Escobar CM, Crowley CS (2010) Short N-terminal sequences package proteins into bacterial microcompartments. Proc. Natl. Acad. Sci. USA 107: 7509–7514.

23. De la Fuente IM (2013) Metabolic Dissipative Structures. In: Systems Biology of Metabolic and signaling Networks: Energy, Mass and Information Transfer (eds. MA Aon, V Saks & U Schlattner). Springer Books. New York. (in press)

24. De la Fuente MI (2010) Quantitative Analysis of Cellular Metabolic Dissipative, Self-Organized Structures. Int. J. Mol. Sci. 11(9): 3540-3599.

25. De la Fuente MI, Cortes J, Perez-Pinilla M, Ruiz-Rodriguez V, Veguillas J (2011) The metabolic core and catalytic switches are fundamental elements in the self-regulation of the Systemic Metabolic Structure of Cells. Plos One 6:e27224.

26. Lloyd, D. Murray, D.B. (2005) Ultradian metronome: timekeeper for orchestration of cellular coherence. Trends Biochem. Sci. 7:373–377.

27. Getty-Kaushik L, Richard AM, Corkey BE 2005 Free fatty acid regulation of glucose-dependent intrinsic oscillatory lipolysis in perifused isolated rat adipocytes. Diabetes 54(3):629-37.

28. Rosenspire AJ, Kindzelskii AL, Petty H, (2001) Pulsed DC electric fields couple to natural NAD(P)H oscillations in HT-1080 fibrosarcoma cells. Journal of Cell Science 114.1515-1520.

29. Marquez S, Crespo P, Carlini V, Garbarino-Pico E, Baler R, et al. (2004) The metabolism of phospholipids oscillates rhythmically in cultures of fibroblasts and is regulated by the clock protein PERIOD 1. FASEB Journal, 18: 519-521.

30. Holz GG, Emma Heart E, Leech CA (2008) Synchronizing Ca2+ and cAMP oscillations in pancreatic beta cells: a role for glucose metabolism and GLP-1 receptors? Am J Physiol Cell Physiol 294:c4-c6.

31. Ainscow EK, Mirsham S, Tang T, Ashford MLJ, Rutter GA (2002) Dynamic imaging of free cytosolic ATP concentration during fuel sensing by rat hypothalamic neurones: evidence for ATPindependent control of ATP-sensitive K+ channels. Journal of Physiology 544: 429-445.

32. Zhaojun XU, So-ichi Y, Kunio T (2004) Gts1p stabilizes oscillations in energy metabolism by activating the transcription of TPS1 encoding trehalose-6-phosphate synthase 1 in the yeast Saccharomyces cerevisiae. Biochem. J. 383: 171–178.

33. Rengan R, Omann GM (1999) Regulation of Oscillations in Filamentous Actin Content in Polymorphonuclear Leukocytes Stimulated with Leukotriene B4 and Platelet-Activating Factor. Biochemical and Biophysical Research Communications 262: 479–486.

34. Shankaran H, Ippolito DL, Chrisler WB, Resat H, Bollinger K, et al, (2009) Rapid and sustained nuclear–cytoplasmic ERK oscillations induced by epidermal growth factor. Molecular Systems Biology 332:1-13.

35. Zhaojun XU, So-ichi Y, Kunio T (2004) Gts1p stabilizes oscillations in energy metabolism by activating the transcription of TPS1 encoding trehalose-6-phosphate synthase 1 in the yeast Saccharomyces cerevisiae. Biochem. J. 383: 171–178

36. Hans MA, Heinzle E, Wittmann Ch (2003) Free intracellular amino acid pools during autonomous oscillations in Saccharomyces cerevisiae. Biotechnol Bioeng. 82(2):143-151.

37. Hartig K, Beck E (2005) Endogenous Cytokinin Oscillations Control Cell Cycle Progression of Tobacco BY-2 Cells Plant Biology 7(1): 33-40.

38. Hungerbuehler AK, Philippsen P, Gladfelter AS (2007) Limited Functional Redundancy and Oscillation of Cyclins in Multinucleated Ashbya gossypii Fungal Cells Eukaryot Cell. 6(3): 473–486.

39. Shaul O, Mironov V, Burssens S, Van Montagu M, Inze D (1996) Two Arabidopsis cyclin promoters mediate distinctive transcriptional oscillation in synchronized tobacco BY-2 cells. Proc Natl Acad Sci. 93(10): 4868–4872.

40. Chabot JR, Pedraza JM, Luitel P, van Oudenaarden A (2007) A Stochastic gene expression out-of-steady-state in the cyanobacterial circadian clock. Nature. 450:1249-1252.

41. Tian B, Nowak DE, Brasier AR (2005) A TNF-induced gene expression program under oscillatory NF-κB control. BMC Genomics. 6:137.

42. Tonozuka H, Wang J, Mitsui K, Saito T, Hamada Y et al. (2001) Analysis of the Upstream Regulatory Region of the GTS1 Gene Required for Its Oscillatory Expression. J. Biochem. 130:589-595.

43. Klevecz RR, Bolen J, Forrest G, Murray DB (2004) A genomewide oscillation in transcription gates DNA replication and cell cycle. Proc Natl Acad Sci. 101(5):1200-1205.

44. Lange G, Mandelkow EM, Jagla A, Mandelklow E (2004) Tubulin oligomers and microtubule oscillations Antagonistic role of microtubule stabilizers and destabilizers. FEBS. 178:61-69.

45. Placantonakis DG, Welsh JP (2001) Two distinct oscillatory states determined by the NMDA receptor in rat inferior olive J. Physiol. 534:123-140.

46. De Forest M, Wheeler CJ (1999) Coherent Oscillations in Membrane Potential Synchronize Impulse Bursts in Central Olfactory Neurons of the Crayfish J Neurophysiol 81:1231-1241.

47. Sánchez-Armáss S, Sennoune SR, Maiti D, Ortega F, Martínez-Zaguila R (2006) Spectral imaging microscopy demonstrates cytoplasmic pH oscillations in glial cells Am J Physiol Cell Physiol 290:C524-C538.

48. Lloyd D, Eshantha L, Salgado J, Turner MP, Murray DB (2002) Respiratory oscillations in yeast: clock-driven mitochondrial cycles of energization.FEBS Lett. 519(1-3):41-44.

49. Danù S, Sùrensen PG, Hynne F (1999) Sustained oscillations in living cells Nature. 402:320-322.

50. Ishii K, Hirose K, Iino M (2006) Ca2+ shuttling between endoplasmic reticulum and mitochondria underlying Ca2+ oscillations EMBO 7:390-396.

51. Jules M, Francois J, Parrou JL (2005) Autonomous oscillations in Saccharomyces cerevisiae during batch cultures on trehalose FEBS Journal 272:1490-1500.

52. Getty L, Panteleon AE, Mittelman SD, Dea MK, Bergman RN (2000) Rapid oscillations in omental lipolysis are independent of changing insulin levels in vivo. J Clin Invest 106: 421-430.

53. Klevecz RR, Murray DB (2001) Genome wide oscillations in expression – Wavelet analysis of time series data from yeast expression arrays uncovers the dynamic architecture of phenotype. Molecular Biology Reports 28: 73-82.

54. Brodsky V, Boikov PY, Nechaeva NV, Yurovitsky YG,Novikova TE, et al. (1992) The rhythm of protein synthesis does not depend on oscillations of ATP level. Journal of Cell Science 103: 363-370.

55. Kindzelskii AL, Zhou MJ, Haugland PR, Boxer A L, Petty RH (1998) Oscillatory Pericellular Proteolysis and Oxidant Deposition During Neutrophil Locomotion. Biophysical J. 74: 90–97.

56. Fuentes JM, Pascual MR, Salido G, Soler JA (1994) Oscillations in rat liver cytosolic enzyme activities of the urea cycle. Archives Of Physiology and Biochemistry. 102(5):237-241.

57. Wittmann C, Hans M, Van Winden AW, Ras C, Heijnen JJ (2005) Dynamics of intracellular metabolites of glycolysis and TCA cycle during cell-cycle-related oscillation in Saccharomyces cerevisiae. Biotechnol Bioeng. 89: 839-847.

58. Aon MA, Roussel MR, Cortassa S, O’Rourke B, Murray DB, et al. (2008) The scale-free dynamics of eukaryotic cells. Plos One 3: e3624, 1-12.

59. Garmendia-Torres C, Goldbeter A, Jacquet M Nucleocytoplasmic (2007) Oscillations of the Yeast Transcription Factor Msn2: Evidence for Periodic PKA Activation. Current Biology 17: 1044-1049.

60. Barril EF, Potter AR (1968) Systematic Oscillations of Amino Acid Transport in Liver from Rats Adapted to Controlled Feeding Schedules. J NUTMTION 95: 228-237.

61. Møller AC, Hauser MJB, Olsen LF (1998) Oscillations in peroxidase-catalyzed reactions and their potential function in vivo, Biophysical Chemistry 72: 63-72.

62. Chiam HK, Rajagopal G (2007) Oscillations in intracellular signaling cascades. Phys Rev E. 75: 061901.

63. Smrcinová M, Sørensen PG, Krempasky J, Ballo P (1998) Chaotic oscillations in a chloroplast system under constant illumination, International Journal of Bifurcation and Chaos 8: 2467-2470.

64. Murray D, Beckmann M, Kitano H (2007) Regulation of yeast oscillatory dynamics Proceedings of the National Academy of Sciences, 104(7):2241-2246.

65. Tu BP, Kudlicki A, Rowicka M, McKnight SL (2005) Logic of the yeast metabolic cycle: Temporal compartmentalization of cellular processes. Science 310: 1152–1158.

66. Lloyd D, Murray DB (2006) The temporal architecture of eukaryotic growth. FEBS Lett. 580:2830–2835.

67. Klevecz RR, Bolen J, Forrest G, Murray DB (2004) A genomewide oscillation in transcription gates DNA replication and cell cycle. Proc Natl Acad Sci. 101(5):1200-1205.

68. Oliva A, Rosebrock A, Ferrezuelo F, Pyne S, Chen H et al (2005) The cell cycle-regulated genes of Schizosaccharomyces pombe, PLoS Biol. 3:1239–1260.

69. Aon MA, Cortassa S, O’Rourke B (2006) The fundamental organization of cardiac mitochondria as a network of coupled oscillators. Biophys. J. 91:4317–4327.

70. Roussel MR, Ivlev AA, Igamberdiev AU (2006) Oscillations of the internal CO2 concentration in tobacco leaves transferred to low CO2. J. Plant Physiol. 34:1188–1196.

71 Berridge MJ, Galione A (1988) Cytosolic calcium oscillators. Faseb. J. 2:3074–3082.

72. Brodsky, VY (2006) Direct cell-cell communication: A new approach derived from recent data on the nature and self-organisation of ultradian (circahoralian) intracellular rhythms. Biol. Rev. Camb. Philos. Soc. 81:143–162.

73. Dekhuijzen A, Bagust J (1996) Analysis of neural bursting: Nonrhythmic and rhythmic activity in isolated spinal cord. J. Neurosci. Methods 67: 141–147.

74. Olsen LF, Degn H (1985) Chaos in biological systems. Q. Rev. Biophys. 18:165–225.

75. De la Fuente MI, Cortes JM (2012) Quantitative Analysis of the Effective Functional Structure in Yeast Glycolysis. PLoS ONE 7(2): e30162.

76. Goldbeter A (2002) Computational approaches to cellular rhythms. Nature 420:238–245.

77. Goldbeter A (2007) Biological rhythms as temporal dissipative structures. Advances in Chemical Physics 135:253-295.

78. Nicolis G, Prigogine I (1977) Self-organization in nonequilibrium systems. From dissipative structures to order through fluctuations. Wiley. New York.

79. Ebeling W, et al (1986) Selforganization by non-linear irreversible processes. Ebeling, W, Ulbricht H. (eds). Springer-Verlag. Berlin.

80. De la Fuente IM (2013) Metabolic Dissipative Structures. In: Systems Biology of Metabolic and signaling Networks: Energy, Mass and Information Transfer (eds. MA Aon, V Saks & U Schlattner). Springer Books. New York. (in press)

81. Saks V, Dzeja P, Schlattner U, Vendelin M, Terzic A, et al. (2006) Cardiac system bioenergetics: metabolic basis of the Frank-Starling law. J Physiol. 571:253–273.

82. Monge C, Beraud N, Kuznetsov AV, Rostovtseva T, Sackett D (2008) Regulation of respiration in brain mitochondria and synaptosomes: Restrictions of ADP diffusion in situ, roles of tubulin, and mitochondrial creatine kinase. Mol. Cell. Biochem. 318:147–165.
