# Supplementary material for: Attractor Metabolic Networks
Source: PLoS One. 2013 Mar 15;8(3):e58284. doi: 10.1371/journal.pone.0058284 (PMC3598861; doi:10.1371/journal.pone.0058284)
Supplement: Supporting Information S3 — Initial conditions of the 18 metabolic subsystems. (DOC) [file pone.0058284.s004.doc]

**Supporting Information S3.**

**Initial conditions of the 18 metabolic subsystems**

| **MSb** | **Initial Conditions** | | |
| --- | --- | --- | --- |
| 1 | .19 | .02 | .59 |
| 2 | .04 | .02 | .44 |
| 3 | .60 | .35 | .68 |
| 4 | .98 | .81 | .28 |
| 5 | .01 | .29 | .01 |
| 6 | .42 | .69 | .99 |
| 7 | .51 | .01 | .49 |
| 8 | .08 | .76 | .13 |
| 9 | .65 | .33 | .36 |
| 10 | .13 | .13 | .94 |
| 11 | .87 | .03 | .08 |
| 12 | .45 | .26 | .09 |
| 13 | .71 | .12 | .25 |
| 14 | .09 | .67 | .03 |
| 15 | .69 | .61 | .11 |
| 16 | .98 | .25 | .87 |
| 17 | .83 | .61 | .40 |
| 18 | .99 | .61 | .10 |
